# Molecular and functional characterization of GMP-manufactured neural stem cells and their extracellular vesicles for innovative therapeutic applications

**DOI:** 10.1186/s13287-026-04904-x

**Published:** 2026-01-09

**Authors:** Martina Guzzetti, Letizia Mezzasoma, Davide Chiasserini, Lara Macchioni, Magdalena Davidescu, Alessandro di Michele, Marco Gargaro, Nicola Di-Iacovo, Giorgia Manni, Gianmarco Muzi, Ilaria Proietti, Giuseppina Bevacqua, Eleonora Becattini, Carlo Conti, Vincenzo Nicola Talesa, Rita Romani, Ilaria Bellezza, Valentina Grespi

**Affiliations:** 1 Department of Medicine and Surgery, University of Perugia, Perugia, Italy; 2 Extracellular Vesicles Network (EV-net) of the University of Perugia, Perugia, Italy; 3 Department of Physics and Geology, University of Perugia, Perugia, Italy; 4 Department of Pharmaceutical Sciences, University of Perugia, Perugia, Italy; 5 Department of Neuroscience, Neurosurgery Unit, Santa Maria Hospital, Terni, Italy; 6 Laboratorio Cellule Staminali, Cell Factory e Biobanca, Santa Maria Hospital, Terni, Italy

**Keywords:** Neural stem cells, Extracellular vesicles, Good manufacturing practice, Immunomodulation, Proteomics

## Abstract

Human neural stem cells (hNSCs) are promising candidates for regenerative medicine due to their self-renewal capacity, differentiation potential, and ability to modulate inflammation. However, several reports showed that the regenerative properties of stem cells are tied to the extracellular vesicles (EVs) they secrete. This study aimed at characterizing hNSCs produced under Good Manufacturing Practice (GMP) conditions and at elucidating the molecular and functional properties of their secreted extracellular vesicles (hNSC-EVs). hNSCs were first assessed for proliferation, and differentiation potential, showing a stable growth profile and expression of neural stem cell markers. High-resolution proteomic analysis identified over 5000 proteins, with about 40% overlap with previous NSCs studies. hNSCs expressed mostly markers for different cell lineage precursors. The molecular characterization of hNSC-derived EVs (hNSC-EVs) showed a size distribution, as measured by nanoparticle tracking analysis, ranging from 140 to 200 nm and an enrichment in EV markers, detected by western blotting. Functional analyses showed that hNSC-EVs, reduce nitric oxide generation and inducible nitric oxide expression in LPS-treated microglial cells and inhibit caspase-1 activation in monocytic cell models through uptake-dependent and independent mechanism, respectively. Our findings show that hNSC possess a strong stemness signature and secrete EVs with immunomodulatory properties, suggesting the worth of hNSC-EVs as either alternative to cell-based therapies or primer to boost anti-inflammatory properties of hNSCs in the treatment of neurological disorders.

**Supplementary Information:**

The online version contains supplementary material available at 10.1186/s13287-026-04904-x.

## Background

Neural stem cells (NSCs) are self-renewing and multipotent cells of the central nervous system (CNS) that give rise to neuron-glial cells during embryogenesis and in adulthood, although only in a few neurogenic niches [1]. NSCs exert their beneficial effects not only by cell replacement but also by immunomodulation and trophic support via paracrine mechanisms and cell-to-cell interaction, which promotes neuroprotection and tissue repair by re-establishing the functional interactions between neural and glial cells [2]. Therefore, there has been considerable interest in NSCs-based therapeutic approaches for various CNS diseases. It is worth noticing that, as with any other medicine, cell-based medical products that are legally classified as advanced therapy medicinal products, should be prepared in compliance with good manufacturing practice (GMP) regulations. Several studies have shown promising results for human NSCs (hNSCs) therapies in chronic neurological diseases such as amyotrophic lateral sclerosis (ALS) and multiple sclerosis (MS) [3–7]. Despite the positive outcomes of cell-based therapies, there are still many challenges and limitations for the translation of stem cells therapies into clinical practice, including issues related to scalability, safety, and regulatory considerations [8]. To increase the translational success of stem cell-based therapies, numerous preclinical studies have suggested that the use of cell-free products such as stem cell secretome, which comprises stem cell-derived soluble factors and extracellular vesicles (EVs), may be a safer option and an alternative therapeutic strategy [8].

Among secretome components, EVs are of particular interest, because they can shape tissue phenotype by mediating cell-to-cell communication. EVs, released by almost all cell types, are composed of a phospholipid bilayer in which transmembrane proteins are immersed and luminally transport nucleic acids, proteins and metabolites that represent EVs cargo. The main function of EVs is intercellular communication. Indeed, EVs transport their cargo to recipient cells which, in turn, modify their behavior according to the received signal [9]. In this light, EV-transported enzymes are transferred to the recipient cells which acquire new or increased capability to convert specific substrates, as in the case of glycolytic enzymes transferred to cardiomyocytes to improve their energy producing ability [10]. Intriguingly, EVs also function as independent metabolic units which, provided that the substrates and the membrane transporters are available, can exert their effects into the extracellular space, apporting direct modification to tissue microenvironment [10–12]. EVs also exert immunomodulatory functions by inducing immunosuppressive responses. Besides their historical involvement in the hiding of sperm cells to the female genital tract immune system to guarantee fecundation [13], they have been found to play a key role in in vitro and in vivo models of neurodegenerative disease by attenuating glial inflammation [14]. It has been also shown that EVs can modulate inflammasome activation in immune cells [12, 15] and reprogram inflammatory conventional dendritic cells (cDC2s) cells towards a tolerogenic phenotype, promoting protection against neuroinflammation in experimental models of MS [16].

A key factor for the application of EVs in neurological disorders therapy is their ability to cross the blood-brain barrier (BBB), transporting functional cargo protected by the EVs membrane, and interacting with specific target cells through surface proteins and lipids [17]. Crucially, their effect seems to be exerted in both CNS and in the periphery [16, 18–20]. Another factor that may facilitate EVs clinical applications with respect to stem cells is linked to their small size and acellular nature that may enable systemic or intranasal delivery with favourable biodistribution and low immunogenicity [16]. Finally, if the stem cells are already produced under Good Manufacturing Practice (GMP) conditions, the isolation protocols can be readily adapted to yield GMP-grade vesicles, facilitating regulatory approval and clinical translation [4]. Given the growing interest in the potential application of EVs for the treatment of neurodegenerative diseases, there is a clear need for an in-depth characterization of the cell of origin and of the functional EVs properties. Based on these premises, this study aimed to thoroughly characterize hNSCs produced under GMP conditions and analyze the EVs secreted by these cells at both molecular and functional levels to explore their potential for innovative therapeutic applications.

## Materials and methods

### Materials

Unless otherwise stated, all used reagents are sourced from Merck, Darmstadt, Germany.

### hNSCs culture

The hNSCs used in this study were obtained from fetal brain tissue derived from foetuses that underwent miscarriage or natural in utero death. The collection of the fetal neural tissues was authorized by the relevant Ethical Committee (Prot. N. 01/CE 25th January, 2012). Each donor provided written informed consent to the tissue collection only after the fetus death. hNSCs were produced and characterized, as previously described [21], in the Cell Factory of the Santa Maria Hospital (Terni, Italy), authorized by the Italian Medicines Agency (AIFA). The methodology applied to isolate, expand, characterize, and cryopreserve the lines is based on the neurosphere assay [3]. hNSC from six different batches were used in the study.

hNSCs were seeded at 10,000 cells/cm^2^ in pre-conditioned media (NeuroCult-XF Proliferation Medium kit, Stem Cell Technologies, Vancouver, Canada), 0,4 UI/ml of heparin (MedicItalia, Milan, Italy), 20 ng/ml epidermal growth factor (EGF) (PeproTech, Cranbury, United States), and 10 ng/ml basic fibroblast growth factor (bFGF) (PeproTech, Cranbury, United States) and incubated at 5% O_2_, 5% CO_2_ and 37 °C for 7–10 days monitoring their growth as neurospheres until they reach a diameter of 100 μm. Neurospheres were mechanically dissociated to obtain a single-cell suspension, and cells were seeded as described for at least five passages. The growth curve was calculated using the formula:

$$\:y\left(P\right)=\:\frac{c}{b}\cdot\:y(P-1)$$

where: P is the passage number, c represents the number of cells obtained at passage P; b represents the number of cells seeded in the previous passage (passage P-1); y(P-1) represents the number of cells obtained at the previous passage (for the first passage y(P-1) = number of seeded cells). Live cells number was quantified over 5 passages using a Burker chamber and the standard trypan blue exclusion method. The slope of the curve was considered the indicator of the cell potency.

After in vitro culture passages, karyotype analysis and SNP array had been performed to evaluate the maintenance of genomic stability. Data showed no structural or numeric abnormalities, and no balanced microrearrangements.

### Clonal efficiency test

Approx. 100 μm-sized neurospheres were mechanically dissociated to obtain a single-cell suspension and seeded in a 24 flat-bottom well plate coated with Poly-L-lysine. At a density of 1000 cells/cm^2^ and 500 cells/cm^2^ and incubated at 5% O_2_, 5% CO_2_ and 37 °C for 7 days. At the end of the incubation, the number of neurospheres with a diameter of 50–100 μm were counted. The percentage of clones was calculated as the ratio between the total cell seeded and the number of neurospheres obtained.

### Immunohistochemistry (IHC) of paraffin-embedded neurospheres

Neurospheres were fixed in 10% paraformaldehyde [22] (PFA), washed in HBSS (Thermo Fisher Scientific, Waltham, United States) and included in 2% agarose and, after dehydration, embedded in paraffin and sliced in 3 μm thick sections with microtome (Leica Biosystem, Milan, Italy). Immunohistochemical staining was performed using a Roche Ventana Benchmark Ultra automated slide stainer (Ventana Medical Systems, Roche, Rotkreuz, Switzerland) with the OptiView DAB IHC Detection Kit (Roche, Rotkreuz, Switzerland). After incubation with the indicated primary antibodies, nuclei were counterstained Hematoxylin II (Roche, Rotkreuz, Switzerland) and Bluing Reagent (Roche, Rotkreuz, Switzerland).

### Differentiation test

For the differentiation test, hNSCs, seeded on glass coverslips coated with Cultrex (Biotechne, Minneapolis, United States) at a density of 30.000 cells/cm^2^, were cultured in medium devoid of EGF and in the presence of bFGF for 3 days at 37 °C in a 5% O_2_, 5% CO_2_ atmosphere. Medium was hence replaced with medium supplemented with 2% FBS, w/o growth factors, and incubated for 7 days. Finally, hNSCs were fixed with 4% PFA for 1 h and stored in PBS at 2–8 °C until immunostaining. The presence of neurons, astrocytes, and oligodendrocytes in the progeny of hNSCs was evaluated by immunostaining with antibodies listed in additional file 1. A total of 2000 nuclei were counted in at least five randomly selected microscopic fields for each experimental replicate. The percentage of differentiated cells belonging to the three neural lineages (neurons, astrocytes, and oligodendrocytes) was calculated based on the total number of nuclei recorded.

### PCR analyses

Total RNA was extracted from hNSCs (5 × 10^6^ cells) using the Ribospin II Kit (GeneAll, Seoul Korea) following the manufacturer’s instructions and cDNA generation was performed with 1 µg of RNA using the PrimeScript™ RT reagent Kit (TakaraBio, Kusatsu, Japan), according to the manufacturer’s instructions. To evaluate expression levels of APL, KLF4, OCT4, SOX2 quantitative PCR amplifications were performed using SensiFast SYBR kit (Meridian Bioscience Inc, Cincinnati, USA) according to the manufacturer’s instructions. GAPDH was used as endogenous control. Primers are listed in additional file 2.

### Extracellular vesicle isolation

EVs were isolated from the conditioned media of hNSCs cultured for 7–10 days according to MISEV guidelines [23]. Cell culture supernatants (conditioned media) were collected, and hNSC-EVs were isolated by differential centrifugation. In detail, the conditioned media were subjected to centrifugation at 800×g for 5 min to remove cells, followed by centrifugation at 2,500 × g for 30 min at 4 °C to remove cell debris. The supernatant was then ultracentrifuged in sterile ultracentrifuge tubes at 100,000×g for 1 h using the SW41 rotor (Beckman Coulter, Pasadena, CA, USA) and the pellet was washed with 0.2 μm filtered PBS for 1 h at 100,000 × g and resuspended in 0.2 μm filtered PBS added with 1% penicillin-streptomycin.

For the determination of EVs markers by Western blotting, according to MISEV2018 guidelines [23], an enrichment step by polyethylene glycol precipitation was applied [24]. In brief, after the 800 × g and 2500 × g centrifugation steps, an equal volume of 2 x PEG 6000 solution (in filtered PBS and 4% sodium chloride) was added to the conditioned medium to obtain a final concentration of 12% PEG. Samples were incubated overnight at 4 °C under constant rotation and then ultracentrifuged at 100,000 × g for 1 h. The resulting pellets were resuspended in Laemmli buffer (60 mmol/L Tris-HCl, pH 6.8, 2% SDS, 10% glycerol, 1% Bromophenol Blue, 1.5% β-mercaptoethanol) for Western blot analysis.

In selected experimental conditions, the following procedure was applied. The EV-depleted conditioned medium (hNSC-CM w/o EVs) was obtained according to the methods described above. The non conditioned medium (hNSC-medium), the hNSC conditioned medium depleted of EVs (hNSC-CM w/o EVs), and complete conditioned media (hNSC-CM w EVs) were analyzed by NTA and then concentrated 9x with 3 kDa cut-off ultrafilters (Amicon^®^ Ultra Centrifugal Filter, 3 kDa MWCO).

### Nanoparticle tracking analysis (NTA)

Nanoparticle Tracking Analysis was performed to determine particle size and concentration. EVs were vortexed for 1 min and loaded into the NS300 NanoSight instrument (Malvern Instruments Ltd, Malvern, UK) according to the supplier’s instructions. Five videos of 60 s were acquired and processed using NTA3.4 software. Particles in Brownian motion were tracked, and their hydrodynamic diameter was calculated using the Stokes-Einstein equation.

### Analysis by scanning electron microscopy (SEM)

EVs were fixed in 1% paraformaldehyde for 30 min at room temperature, washed with filtered distilled water, sedimented on coverslips, and allowed to dry at room temperature. SEM images were obtained using a field emission scanning electron microscope (Zeiss LEO 1525; Thornwood NY, USA) with chromium metallization using a high-resolution ES-Quorum 150 T apparatus (24 s, sputter at 240 mA current). Chromium thickness was ~ 10 nm [12].

### THP-1 and BV2 cell culture and treatments

Human THP-1 monocytes were purchased from American Type Culture Collection (ATCC, USA) and routinely maintained at 37 °C in a 5% CO_2_ humidified atmosphere in RPMI 1640 supplemented with 10% heat-inactivated FBS, L-glutamine, 1 mmol/L sodium pyruvate, non-essential amino acids, 1% of penicillin/streptomycin. Murine microglial BV2 cells were a kind gift from Prof R. Donato (University of Perugia). Cells were maintained in RPMI 1640 supplemented with 10% heat-inactivated FBS at 37 °C in a 5% CO_2_ humidified atmosphere.

THP-1 cells (3 × 10^6^ cells/well) were plated in 6-well culture dishes and after 1 h were pretreated with 1 × 10^3^ hNSC-EVs/cell for 1 h. THP-1 cells were subsequently primed with 10 µg/ml LPS for 20 min and then activated with 5 mmol/L ATP for 40 min. In independent experiments, THP-1 cells were treated with 500 nmol/L ZM-241385 (4-(2-[7-A mino-2-(2-furyl) [1, 2, 4]triazolo[2,3-*a*] [1, 3, 5]triazin-5-ylamino]ethyl)phenol, (Tocris, Bio-techne, Minneapolis, MN, USA) 1 h before treatments. Vehicle-treated cells (DMSO) did not show any significant difference with respect to untreated control cells; therefore, all the relative treatments were compared to these latter controls. At the end of the treatments, total cell lysates were prepared using RIPA buffer with protease and phosphatase inhibitors (Roche, Rotkreuz, Switzerland).

BV2 cells were seeded at a density of 6.5 × 10^4^ cells/cm^2^, after 24 h cells were pretreated with 1 × 10^3^ hNSC-EVs/cell for 1 h and subsequently exposed to 10 µg/ml LPS for 24 h [25]. Total cell lysates were obtained by resuspending cell pellets in boiling Lamely sample buffer (1 × 10^7^ cells/ml).

The number of EVs used for the treatment of THP-1 cells was chosen based on data obtained with EVs of different origin [12]. As for BV2, the amount was chosen based on preliminary experiments in which EV number/cell ranged from 5 × 10^2^ to 5 × 10^3^.

### Cell viability

BV2 cells viability was assessed by the conventional MTT (3-[4,5-dimethylthiazol-2-yl]− 2,5-dephenyl tetrazolium bromide) reduction assay. Briefly, at the end of the treatment, cells were exposed to MTT (final concentration 0.5 mg/ml) for 2 h, then the medium was removed, and formazan salts were dissolved in DMSO. The absorbance was measured at 595 nm using a microplate reader (Tecan, Männedorf, Switzerland). Results were expressed as the percentages of reduced MTT, assuming the absorbance of control cells as 100% [25].

### Measurement of NO production

Nitric oxide (NO) production was indirectly determined through the measurement of nitrite, a stable metabolite of NO, by means of Griess reaction. At the end of the treatment cell culture medium was mixed with an equal volume of Griess reagent and after a 20 min incubation at room temperature the absorbance was measured at 550 nm using a microplate reader (Tecan, Männedorf, Switzerland). Results were expressed as percentage of the control, assumed as 100% [25].

### Western blot analysis

For EVs protein analysis, PEG-enriched hNSC-EVs samples were lysed in Laemmli buffer and sonicated four times (5s, 5s break) on ice.

Total cell and EVs lysates were separated by 12% sodium dodecyl sulfate-polyacrylamide gel electrophoresis (SDS-PAGE) and transferred to a nitrocellulose membrane. Non-specific binding sites were blocked in 5% milk in TBS-T or Rotiblock buffer (Carl Roth GmbH, Karlsruhe, Germany) for 1 h at room temperature. The membranes were incubated with anti-human primary antibodies overnight at 4 °C. The list of antibodies is reported in additional file 1. After washing with TBS-T, the membranes were incubated with the appropriate HRP-conjugated secondary antibodies for 1 h at room temperature and chemiluminescence was detected using CL Xposure film (Thermo Fisher Scientific, Waltham, MA, USA) or iBright™ CL1500 Imaging System (Thermo Fisher Scientific, Waltham, MA, USA).

### ATP measurement

ATP measurement was performed with an in vitro assay. 20 µg of EVs were incubated in “glycolytic buffer” containing: 10 mmol/L MgCl_2_, 10 mmol/L KCl, 10 mmol/L β-nicotinamide adenine dinucleotide (NAD^+^), 10 mmol/L DTT, 50 µmol/L ADP in PBS. The mixture was preincubated for 10 min at 37 °C, and then added with 5 mmol/L glucose or PBS for the control, for 30 min at 37 °C. The mixture was preincubated for 10 min at 37 °C. ATP amount was evaluated by a Bioluminescent Assay Kit. ATP levels were quantified by a calibrated standard curve [26]. All solutions were sterile and contained penicillin streptomycin.

### Uptake of hNSC-EVs by THP1 and BV2 cells

For the detection of EVs uptake, hNSC-EVs were stained for 30 min with 50 µmol/L DiL (1,1’-Dioctadecyl-3,3,3’,3’-Tetramethylindocarbocyanine Perchlorate (‘DiI’; DiIC18(3)) (Thermo Fisher Scientific, Waltham, MA, USA) and washed by ultracentrifugation for 1 h at 100,000 × g. The pellet was resuspended in PBS supplemented with 1% penicillin/streptomycin solution. THP-1 cells (3 × 10^5^ cells) and BV2 cells (2.5 × 10^4^ cells) were exposed to labeled EVs for 2 h and 24 h, respectively. Cells were fixed in 4% PFA for 20 min at room temperature and actin filaments were stained with Phalloidin (Thermo Fisher Scientific, Waltham, MA, USA) for 30 min at room temperature. Cell nuclei were counterstained with Hoechst 33258 (Thermo Fisher Scientific, Waltham, MA, USA). DiI-labeled cells were imaged using a Nikon Eclipse Ti inverted microscope (Nikon Instruments, Japan) equipped with a CrestOptics X-Light V2 spinning-disk confocal unit. Image acquisition was performed using a 60× oil-immersion objective. Z-stack acquisition parameters, including step size and total stack depth, were controlled using NIS-Elements software (Nikon Instruments), which was also used for generating orthogonal (x–z and y–z) reconstructions. All images within each experimental set were acquired under consistent illumination and exposure conditions to ensure comparability. Raw imaging data were exported in lossless ND2 format and subsequently analyzed using ImageJ/Fiji (National Institutes of Health, USA).

### FACS analyses

hNSC were stained with Alexa Fluor^®^ 647- conjugated anti-Oct4 antibody (Biolegend, San Diego, CA, USA) or anti-KLF4 antibody (LSBiol, Mowry Ave Newark, CA, USA) according to standard protocols. The cells were permeabilized with 0.2% (vol/vol) Triton-X for 30 min at room temperature before staining. Cells were analyzed on an EPICS flow cytometer using the EXPO 32 ADC software (Beckman Coulter, Pasadena, CA, USA) [27].

### Proteomic analysis of hNSCs

Sample preparation was performed according to Macchioni et al. [28], with some modifications. Cell pellets were lysed with RIPA buffer and 100 µg of proteins were precipitated in acetone and then reduced and alkylated in a solution of 6 mol/L Guanidine-HCl, 5 mmol/L TCEP, and 20 mmol/L chloroacetamide. Peptides were obtained by digesting proteins with sequencing-grade endopeptidase Trypsin (Promega, Madison, WI, USA) overnight at 37 °C. Collected peptide mixtures were concentrated and desalted using the Stop and Go Extraction (STAGE) technique on custom made tips. Instruments for LC MS/MS analysis consisted of a NanoLC 1200 coupled via a nano-electrospray ionization source to the quadrupole-based Q Exactive HF benchtop mass spectrometer. Peptide separation was carried out according to their hydrophobicity on a home-made chromatographic column, 75 μm ID, 8 μm tip, 350 mm bed packed with Reprosil-PUR, C18-AQ, 1.9 μm particle size, 120 Angstrom pore size, using a binary buffer system consisting of solution A: 0.1% formic acid and B: 80% acetonitrile, 0.1% formic acid.

For DIA acquisition, runs of 60 min were used for all the samples, with a constant flow rate of 300 nl/ min. After sample loading, the run followed a series of linear gradients, from 7% to 32% buffer B in 45 min, then 5 min and 30 s to reach 45% and 3 min and 30 s to reach 95%. This final step was maintained for 3 min and 30 s. DIA acquisition settings were as follows: MS spectra were acquired using 32 variable windows covering a mass range of 300–1650 m/z. The resolution was set to 60,000 for MS1 and 30,000 for MS2. The AGC was 3 × 10^6^ in both MS1 and MS2, with a maximum injection time of 60 msec in MS1 and 54 msec in MS2. NCE were set to 25%, 27.5%, 30%. After acquisition, raw spectra files were converted into mzXML format via MSconvert [29] for importing into DIA-NN 1.9.1 for database search, protein identification and quantification [30].We used a two-pass search strategy, in which data were first searched with a library-free approach using a UniProtKB FASTA (2024_08, 20,435 sequences) as in-silico database to generate a spectral library from the raw files. In the second step, data were searched again using the spectral library generated from the raw data. The following options were used: min peptide length = 7, max peptide length = 30, FASTA digest for library-free search/library generation; deep learning-based spectra, RTs and IMs prediction; maximum number of variable modifications = 2, modifications = N-term M excision, C carbamidomethylation, Ox(M); neural network classifier = double-pass mode; quantification strategy = high accuracy; precursor false discovery rate (%) = 1.0. Match-between-runs (MBR) mode was enabled in all the analyses. The protein matrix file was loaded into R for processing (v. 4.1.3). MaxLFQ algorithm embedded in diann-R package [30] (https://github.com/vdemichev/diann-rpackage) was used for obtaining protein intensities. Data were filtered for proteins identified in all three replicates, ranked and divided in quintiles according to protein abundance. Each quintile was subjected to overrepresentation analysis using the clusterprofiler R package [31] and the MSigDb gene ontology collection [32]. To derive the list of SC markers and NSC markers we searched for relevant papers and established a list of 56 markers (additional file 3) to obtain a comprehensive overview of the data present in the literature. We then performed overlap analysis with the list of the proteins identified in this work. The list of brain cell type markers was instead downloaded from the Human Protein Atlas (https://www.proteinatlas.org/humanproteome/single+cell) For the overlap analysis with our dataset, a simplified cell type was used, combining inhibitory and excitatory neurons in the class “Neurons” and oligodendrocytes precursor and oligodendrocytes in the class “Oligodendrocytes” (additional file 4). In-house R scripts were used for plotting overlap analysis and ranking proteins according to abundance and stem cell / brain cell markers.

### Statistical analysis

All data, reported as mean ± SD, were analyzed by ordinary one-way ANOVA followed by Tukey’s post hoc test, using GraphPad Prism 9.0 software. p-values < 0.05 were defined as statistically significant.

## Results

### Human neural stem cell cultures characterization

**Fig. 1:**
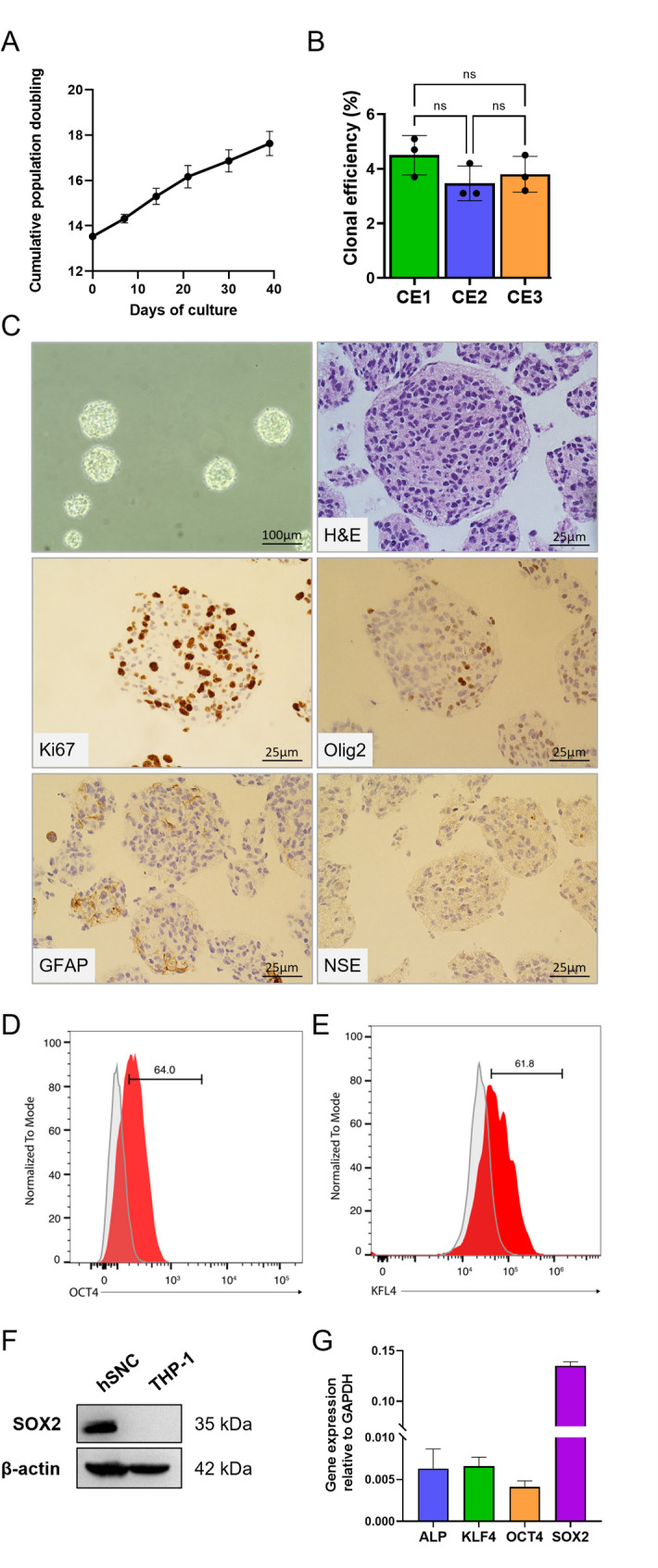
hNSCs characterization.** A** Cumulative growth curve over 5 weeks of culture;** B** Clonal efficiency determined in three different hNSCs batches (CE1: clonal efficiency batch 1; CE2: clonal efficiency batch 2; CE3: clonal efficiency batch 3);** C** Representative images of hNSCs grown as neurospheres after 7 days of culture (magnification 10x); IHC performed on paraffin-embedded hNSCs neurospheres stained for Hematoxylin and Eosin, Ki-67, Olig2, GFAP and NSE (magnification 40x);** D** Expression of OCT4 and** E** KLF4. Representative flow cytometry histograms showing the expression levels of OCT4 and KLF4 in hNSCs. Specific antibody staining is shown in red, and isotype control in grey. The numbers indicate the percentage of positive cells relative to the isotype control;** F** Western blotting analysis of SOX2 in hNSC total cell lysate. THP-1 cells were used as negative control; β-actin was used as loading control. The images are representative of one out of three separate experiments;** G** Gene expression levels of ALPL, OCT4, KLF4, SOX2 were quantified using the 2^−ΔCt^ method, with GAPDH as the internal control. Data represent mean ± SD from three independent experiments

**Fig. 2:**
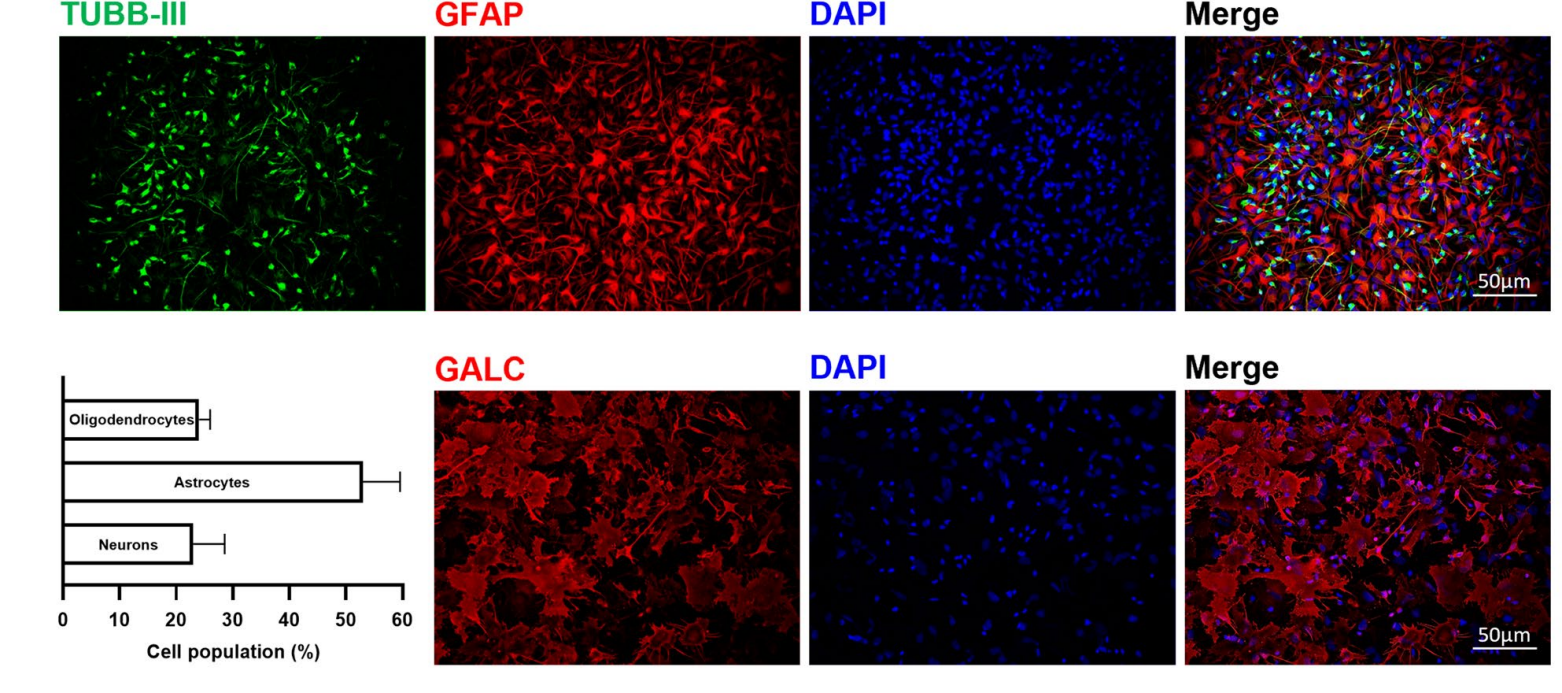
hNSCs differentiation ability. Representative fluorescent images of hNSCs derived neurons (TUBB-III, green), astrocytes (GFAP, red) and oligodendrocytes (GALC, red) at 12 days upon differentiation. All cells are counterstained with nuclear- specific DAPI staining (blue) (Magnification 20x). Images were adjusted for brightness and contrast. Histogram showing the mean percentages of human neural stem cells differentiated into oligodendrocytes, neurons, and astrocytes. Data are presented as mean ± SD from three independent batches

hNSCs were extensively characterized by a consolidated paradigm to ensure their identity including assessment of their self-renewal activity, clonal efficiency and differentiation capacity. Cells growth kinetic remained stable over time (curve slope = 0.107 ± 0.016; optimal range 0.025–0.165 [33]) and no change in cumulative growth was observed over 5 weeks of culture (Fig. 1A); clonal efficiency assay showed that cells retained the ability to form clonal neurospheres under stringent culture conditions (optimal value > 1% [33]) (Fig. 1B). Immunohistochemistry (IHC) staining allowed to evaluate the cytoarchitecture of the neurospheres and the protein expression within (Fig. 1C). Ki-67, a well-described biomarker of cell proliferation, was strongly detected in neurospheres, demonstrating active proliferation, which is a key feature of stem cells [34]. Instead, only a small percentage of cells were positive for the glial restricted precursor markers (as Olig2, a transcription factor participating in oligodendrocyte differentiation, and GFAP, the hallmark intermediate filament protein in astrocytes) and for the neuronal precursor markers (NSE, neuron-specific enolase) (Fig. 1C). Our results, shown in Fig. 1D-G, demonstrate that hNSCs expressed the main transcription factors responsible for the maintenance of stemness SOX2 (SRY-box transcription factor 2), OCT4 (octamer-binding transcription factor 4), KLF4 (Krüppel-like factor 4), and ALPL (Alkaline phosphatase) [35]. Finally, we evaluated hNSCs multipotency, i.e. their ability to give rise to the three major neural lineages. This critical feature of hNSCs was determined by in vitro differentiation test, in which the simultaneous presence of neurons (Tubulin III, TUBB III), astrocyte (Glial Fibrillary Acidic Protein, GFAP) and oligodendrocyte (Galactocerebroside, GALC) was detected by immunocytochemical labeling. Quantitative analysis showed that differentiated cells consisted of approximately 53% astrocytes, 23% neurons, and 24% oligodendrocytes (Fig. 2).

### Proteomic characterization of human neural stem cells

**Fig. 3:**
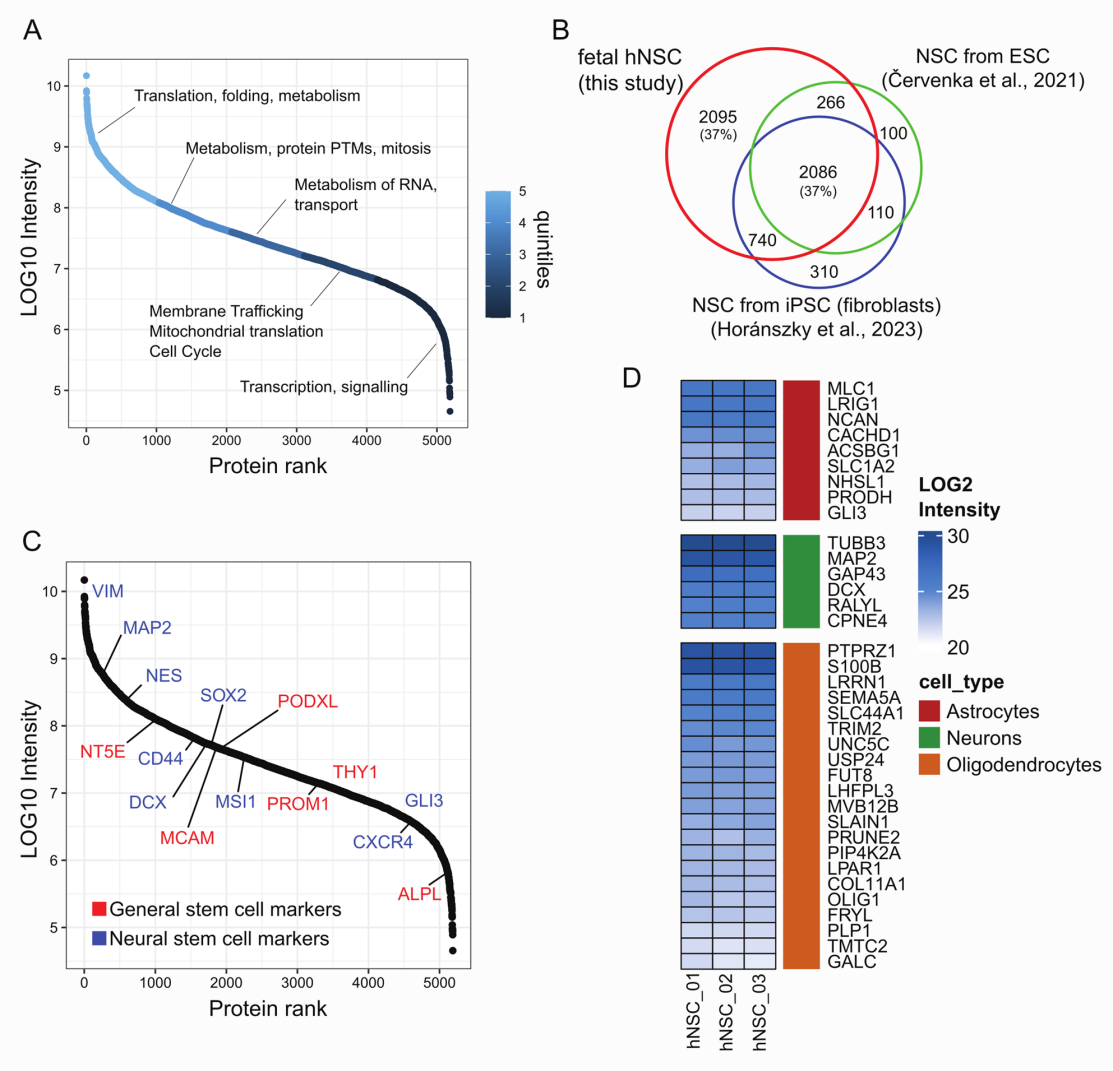
Proteomic analysis of hNSC.** A** Order of magnitude of protein abundance in hNSC proteome. Pathway analysis of quintiles of protein abundances;** B** overlap analysis of hNSC dataset with previously published NSC datasets;** C** Abundance of general stem cell markers and neural stem cell markers in the hNSC proteome;** D** Heatmap of proteins expressed in hNSC clustered according to single cell expression data from the Human protein Atlas

To get an in-depth characterization of hNSCs, we assessed the molecular protein profile of hNSCs by high-resolution mass spectrometry. Proteomic analysis of hNSCs identified 5187 protein groups (additional file 5). Figure 3 shows the characteristics of the hNSCs proteome with respect to general protein abundance and comparison with previous evidence [36, 37]. The proteins identified in hNSCs spanned over 5 orders of magnitude, with a good correspondence between protein abundance and putative functions (Fig. 3A). The identified proteins were divided into quintiles according to their protein intensity (see methods) and overrepresentation analysis was carried out to find biological processes enriched in each quintile. High abundance proteins (1st quintile) were related to processes like translation, protein folding and metabolism, while proteins with lower abundance (5th quintile) were associated with processes like transcription and signaling (Fig. 3A). We then performed overlap analysis to compare the proteome of hNSCs to previously published proteomic datasets related to NSCs. We found that the number of identified proteins in this work was larger than that of two previous studies performed in similar experimental models and with relatively similar proteomic approaches, one analyzing the proteome of NSCs derived from iPSC-derived neural stem cells [36] and one of NSCs spontaneously differentiated from embryonal stem cells [37]. About 37% of the proteins (*n* = 2095) identified in our dataset were not present in either of the two studies, while 37% of the total was identified in all three studies (Fig. 3B). We then searched the literature for general protein markers of SCs (expressed in several stem cell types), and NSCs markers (mainly expressed in NSCs) and looked at their expression level in the hNSCs proteome. We found that the hNSCs expressed relatively high levels of the general stem cell marker NT5E (also called CD73) while the levels of ALPL were much lower (Fig. 3C). Other proteins often characterizing stem cells were also identified, as PODXL and PROM1. For neural stem cells markers, the hNSCs expressed high levels of SOX2, NES, DCX, CXCR4 (also called CD184) and MSI1, all well-known markers of neural stem cells [38, 39]. To further characterize the hNSCs proteome, we downloaded single cell RNA expression data from the Human Protein Atlas [www.proteinatlas.org] to see if in undifferentiated state, the hNSCs expressed protein markers that characterize brain cell types such as astrocytes, neurons and oligodendrocytes. Our hNSCs model expressed proteins which are enriched in neurons (GAP43, MAP2, DCX), oligodendrocytes (S100B, OLIG1, PLP1) and astrocytes (NCAN, SLC1A2). Numerically, the oligodendrocyte enriched proteins were predominant, while expression-wise, the neuronal markers showed the highest expression (Fig. 3D). These results, besides confirming the data of the canonical characterization, extended the knowledge on hNSCs stemness signature.

### Extracellular vesicle isolation and morphological characterization

**Fig. 4:**
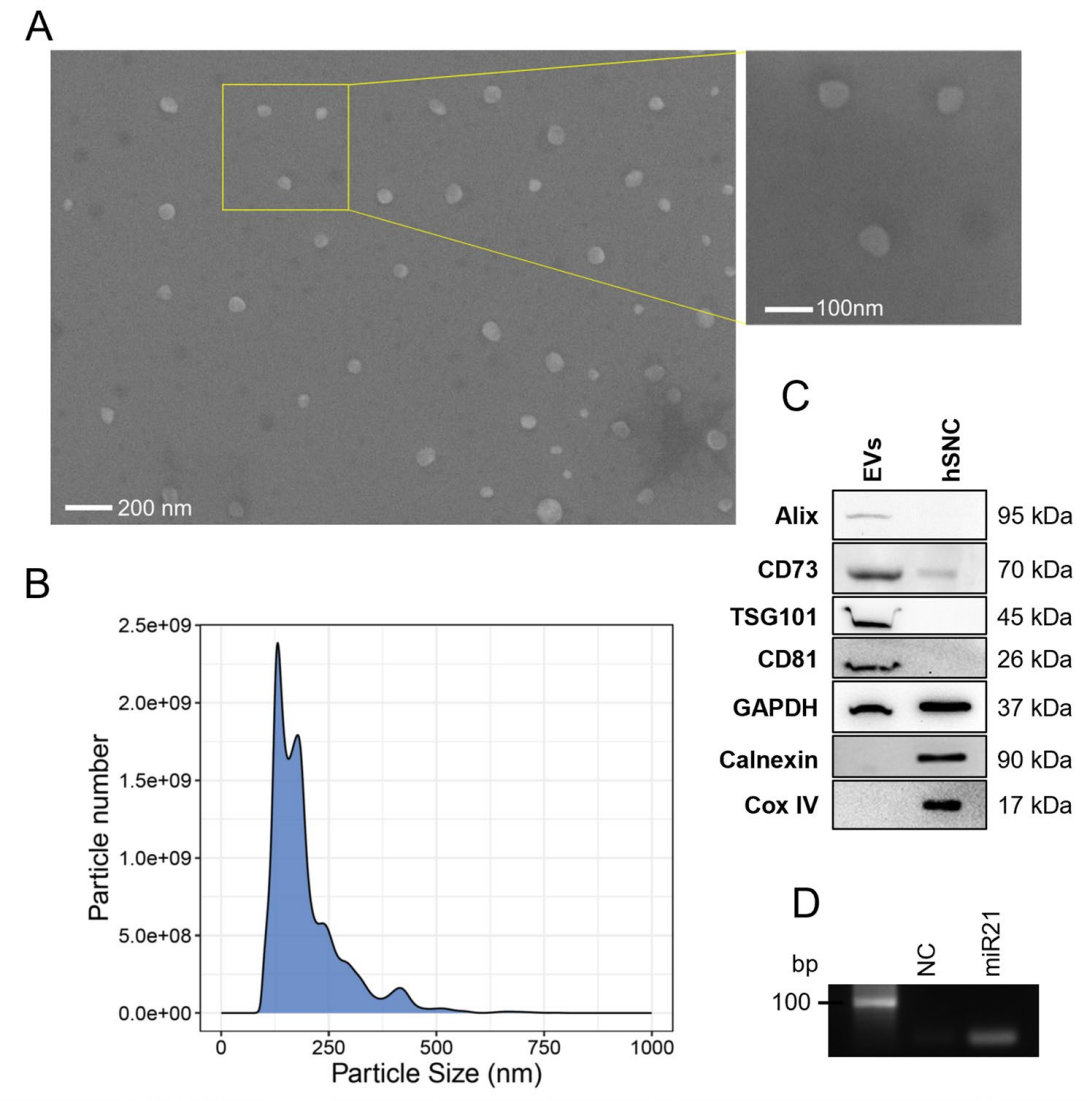
Molecular characterisation of hNSC-EVs. hNSC-EVs were isolated from cell conditioned media by ultracentrifugation;** A** EVs morphology and diameter evaluation by SEM imaging (magnification 50,000 x and 100,000 x);** B** Size distribution of EVs determined by NTA;** C** Characterisation of EVs protein by Western blot. EVs lysates were probed with EVs positive protein markers (Alix, CD81, TSG101, GAPDH), negative protein markers (Calnexin as endoplasmic reticulum marker and COX IV as mitochondrial marker), and CD73. hNSCs cell lysate was used as positive control;** D** Qualitative PCR of miR21 expression in hNSC-EVs

Due to the potential application of stem cell-derived EVs in therapeutic protocols, a molecular and functional characterization of hNSC-EVs is needed. EVs were isolated by a conventional differential centrifugation protocol from the conditioned media of hNSCs, manufactured in GMP conditions, by ultracentrifugation to strike the optimal balance between recovery and specificity. SEM analyses revealed the presence of spherical particles with an average diameter of 77 ± 19 nm (Fig. 4A). NTA analyses showed that hNSC-EVs with a mean hydrodynamic diameter of 197 ± 2 nm (Fig. 4B).

NTA analyses evidenced the presence of two most abundant EVs populations: the predominant group with a mean hydrodynamic diameter of 199 ± 8 nm and a second highly abundant population with a mean hydrodynamic diameter of 140 ± 27 nm (Fig. 4B). Based on the NTA data, we calculated that, at the end of the hNSC subculture period (7–10 days), there is an EVs/cell ratio of 4.18 × 10^3^ ± 2.10 × 10^2^.

The characterization of EVs protein content confirmed the presence of the EVs protein markers Alix, CD81, TSG101 and GAPDH along with the absence of negative protein markers characteristics of specific cellular organelles, such as calnexin (endoplasmic reticulum) and COX IV (mitochondria) (Fig. 4C). Moreover, hNSC-EVs contain CD73, an enzyme detected in the EVs isolated from several tissues and cell types^15^ (http://www.microvesicles.org/) and evidenced by our proteomic analyses, and miR-21 (Fig. 4D), a micro-RNA known to be involved in the modulation of inflammatory cytokine secretion [40].

### Immunomodulatory effects of hNSC-EVs in microglia and immune cells

**Fig. 5:**
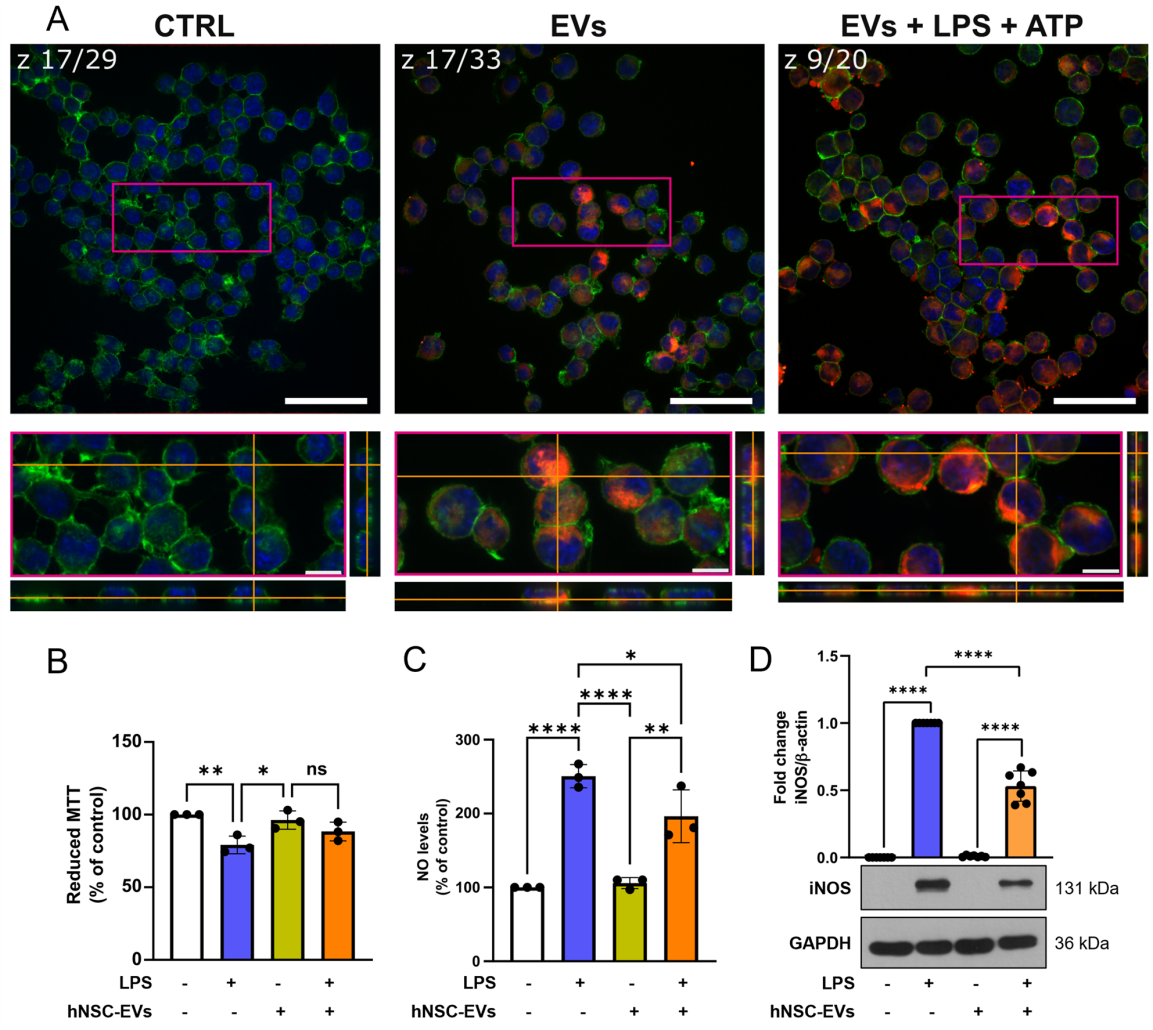
Immunomodulatory effects of hNSC-EVs on BV2 microglial cells. **A** hNSC-EVs uptake by BV2 cells. Cells were treated with DiL-labeled hNSC-EVs in red for 1 h and then exposed for further 24 h with or without LPS, phalloidin in green was used for actin filaments staining and nuclei in blue were counterstained with Hoechst 33258. Upper images show the whole z-stack section (as reported CTRL z:17/29; EVs z:17/33; EVs + LPS z:17/29) with a 50 μm white scale bar. The bottom panels represent the zoomed orthogonal images (fuchsia box) from the z-stack acquisition with a 5 μm white scale bar;** B** Cell viability, detected by MTT assay (absorbance of control cells = 2.2 ± 0.5 was assumed as 100%). Data represent mean ± SD of *n* = 3 independent experiments performed in quadruplicate; **C ** NO production, detected by Griess reagent (absorbance of control cells = 0.048 ± 0.003 was assumed as 100%). Data represent mean ± SD of *n* = 3 independent experiments performed in quadruplicate;** D** Western blotting analysis of iNOS. β-actin was used as loading control. Bars represent the ratio between respective protein and GAPDH band intensity. The images are representative of one out of three separate experiments performed with EVs isolated from different hNSCs batches

To functionally characterize the hNSC-EVs, LPS activated microglia was selected as a model to test hNSC-EVs effects on neuroinflammation, giving its pivotal role in the pathogenesis of neurodegenerative diseases [39]. As shown in Fig. 5A and additional file 6 A, murine BV2 microglial cells internalize hNSC-EVs with cytoplasmic red DiL staining recovered in almost all the analyzed cells either in the presence or in the absence of 10 µg/ml LPS. BV2 exposed to 10 µg/ml LPS [25] showed a slight decrease in cell viability and an increase in NO levels. A 1 h pre-treatment with hNSC-EVs (1 × 10^3^/cell) did not exert cytoprotective effects (Fig. 5B) but caused a statistically significant reduction in NO levels (Fig. 5C) which correlated with a significant decrease in iNOS expression (Fig. 5D).

**Fig. 6:**
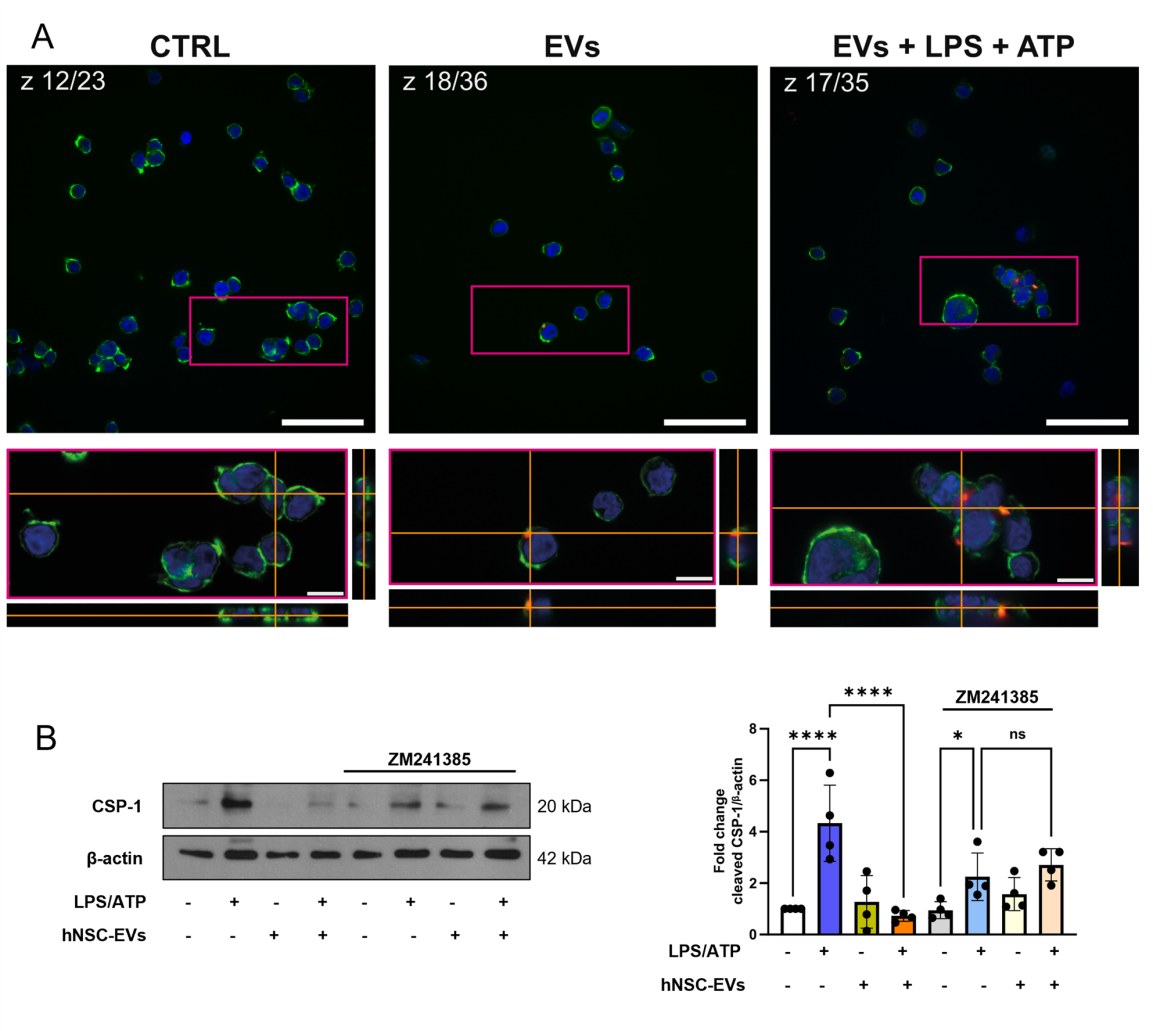
Immunomodulatory effects of hNSC-EVs on THP-1 monocytic cells. **A** hNSC-EVs uptake by THP-1 cells. Cells were treated with DiL-labeled hNSC-EVs in red for 1 h and then exposed for further 1 h with or without LPS + ATP; phalloidin in green was used for actin filaments staining and nuclei in blue were counterstained with Hoechst 33,258. Upper images show the whole z-stack section (as reported CTRL z:12/23; EVs z:18/36; EVs + LPS + ATP z:17/35) with a 50 μm white scale bar. The bottom panels represent the zoomed orthogonal images (fuchsia box) from the z-stack acquisition with a 5 μm white scale bar;** B** Caspase-1 activation. THP-1 cells were pre-treated for 1 h with 1000 hNSC-EVs/cell, subsequently primed for 20 min with 10 µg/ml LPS and then activated for 40 min with 5 mmol/L ATP (LPS). In selected experiments ZM-241,385 (500 nmol/L) was added 1 h before hNSC-EVs treatment; cell lysates were immunoblotted for Caspase-1. The blots were re-probed with mouse anti-β-actin, to confirm equal loading. Representative western blots images are shown. Histogram on the right represents densitometric quantification and indicates the mean ± SD of at least *n* = 3 independent experiments performed with EVs isolated from different hNSCs batches

To confirm the role of hNSC-EVs in neuroinflammation, their effect on the inflammasome, the key player in the neuroinflammation related to neurological disorders [41], was assessed using the human monocytic THP-1 cells, a well-established model for inflammasome activation [42]. A 1-hour pre-treatment of THP-1 cells with hNSC-EVs (1 × 10^3^/cell) before LPS (10 µg/ml, 20 min) and ATP (5 mM, 40 min) stimulation, showed that THP1 cells did not demonstrate a substantial uptake of hNSC-EVs either in the presence or in the absence of LPS + ATP (Fig. 6A and additional file 6D). Nonetheless, hNSC-EVs exert a strong anti-inflammatory effect, by preventing the expression of the active p-20 Caspase-1 fragment, hallmark of inflammasome activation (Fig. 6B), suggesting that the modality of interaction may differ from that of BV2 cells.

We have previously shown that hAFSC-EVs inhibit inflammasome activation by producing adenosine from ATP, which in turn, by activating adenosine A2a receptor on THP-1 cells, inhibits NLRP3-inflammasome platform assembly [12]. The same mechanism was tested for hNSC-EVs. Results show that, when placed in an appropriate glycolytic buffer, 10^6^ hNSC-EVs produce 18.42 ± 2.5 pmol of ATP and that pre-treatment of THP-1 cells for 1 h with the selective adenosine A2a receptor antagonist ZM-241385 (500 nM) prevented hNSC-EVs-induced inhibitory effect on Caspase-1 activation (Fig. 6B). These data suggest that a possible mechanism of the anti-inflammatory effect of hNSC-EVs on THP-1 cells could be via adenosine-autonomously produced by hNSC-EVs.

To ascertain that the observed effects were to be attributed to EVs and not to co-isolated material, conditioned media were concentrated with a 3KDa cut-off ultrafilters to yield a final volume suitable for treating target cells with the desired number of EVs (1000 EVs per cell). To control for effects arising from hNSC medium components, we also assessed the impact of complete, non-conditioned medium. No significant effects were observed upon treatment with either medium (non-conditioned) or EV-depleted conditioned media, confirming that the observed cellular responses are specifically mediated by EVs (additional file 6). Moreover, we did not observe any significant effect of whole conditioned media (not depleted of EVs) on LPS-induced reduction in cell viability and NO production in BV2 cells; this result could be explained by the potential presence of a protein corona that, under in vitro culture conditions, can mask EVs components crucial for their uptake (additional file 6B-C). On the other hand, we observed a partial inhibitory effect exerted by conditioned media (not depleted of EVs) on caspase-1 activation in THP-1 cells (additional file 6E), strengthening our observation that this immunomodulatory effect is uptake-independent and relies on the production of soluble metabolites, an effect that can be independent of the presence of the protein corona.

## Discussion

In this work we have performed a comprehensive characterization of human fetal neural stem cells (hNSCs) and showed that they secrete extracellular vesicles (EVs) with anti-inflammatory properties. Our hNSCs display a staminal signature expressing neural stem cell markers and can differentiate in neurons, oligodentrocytes and microglia, supporting their use in regenerative therapies. EVs secreted by hNSCs attenuate the inflammation induced by acute LPS treatment on in vitro cell models representative of CNS microglial cells and peripheral monocytes, providing a foundation for further investigations as therapeutic agents in neurological and neurodegenerative disorders. Inflammation is recognized as a hallmark of neurodegenerative diseases [43] and the immunomodulatory abilities of EVs might complement or substitute those of hNSCs in therapy. Several attempts have been made to employ NSCs for the treatment of different neurological disorders, including ALS and MS. hNSCs have been already employed in phase 1 clinical trials where they have been administered to ALS or MS patients through unilateral or bilateral injections into the lumbar cord tract [3, 4, 6, 7], or through intraventricular injections [5]. These trials demonstrated procedure safety and, although they were not powered to detect efficacy, also showed that this therapeutic approach did not cause an acceleration of disease progression while in some cases, induced an improvement of clinical score in the first weeks after transplantation [3–7]. In this work we performed an in-depth characterization of hNSCs showing that they maintain a stable growth kinetic and colony forming ability, while being also able to differentiate in the three main cell types, i.e. oligodendrocytes, astrocytes and neurons. Moreover, we found that hNSCs express the Yamanaka transcription factors (SOX2, OCT4, and KLF4) which stringently control developmental signalling pathways to maintain the pluripotency of embryonic stem cells [35]. Our data independent acquisition (DIA) proteomics revealed that hNSCs expressed both general and NSC markers. As for the general markers, CD73 may be involved in the maintenance of the undifferentiated state [44], or in the modulation of the local immune response [45], and ALPL is a key marker of stemness and proliferative capacity [46]. As for neuronal markers, the co-expression of SOX2, NES, DCX, CXCR4, and MSI1 in hNSCs aligns with previous reports in the literature for adult NSCs, confirming their identity and undifferentiated state [38, 39]. The different levels of the identified markers likely reflect their sequential expression during cell differentiation [39], which in turn defines the identity and abundance of different NSCs subpopulations. Proteomic analysis and neurosphere immunolabeling support this hypothesis, suggesting a heterogeneous cell population that includes cells expressing protein markers that characterize different brain cell types. Our data support the possibility that, upon transplantation, hNSCs may promote neural regeneration differentiating into different CNS cells as demonstrated in animal models [47, 48]. Nonetheless, it has been shown that transplanted hNSCs can also modulate the cerebrospinal fluid (CSF) immunological profile toward an anti-inflammatory phenotype [6] supporting the notion that hNSCs exert their effects through multiple mechanisms [47, 48]. Although hNSCs transplantation show a great promise in the treatment of neurological diseases, several safety and regulatory concerns are associated to cell-based therapies including malignant transformation and immune rejection [49, 50]. It has been shown that the efficacy of hNSC, both in animal models and in human, is primarily due to their ability to dampen inflammation, an effect that is elicited through a paracrine mechanism [51], implying that the hNSC secretome can be a rich source of immunomodulatory factors. Within the secretome, EVs are recognized as pivotal immunomodulatory agents in several inflammatory conditions [12, 16, 18, 20]. EVs may pose less concerns in therapeutic applications with respect to SCs. For instance, since EVs are unable to replicate, they are not likely to exert a pro-tumorigenic role [52]. Moreover, compared to cell-based therapy, EV‐based therapy has several advantages including biocompatibility, low immunogenicity, and the ability to cross epithelial barriers, including the BBB [8]. Therefore, hNSC-derived EVs represent a compelling cell-free alternative capable of recapitulating the key immunomodulatory effects of their parental cells while bypassing many limitations of live-cell transplantation [19]. In this context, we isolated and characterized the EVs population secreted by hNSCs to determine whether they could exert general immunomodulatory effects. To obtain an optimal balance between recovery and specificity [9], we isolated hNSCs-EVs by differential ultracentrifugation and obtained a pool of EVs with a mean hydrodynamic diameter of 197 ± 2 nm. SEM analyses revealed the presence of round-shaped particles with a mean diameter of 77 ± 19 nm. The discrepancies in the EVs dimensions observed between SEM and NTA analyses may depend on the fact that the two techniques measure different properties of the sample. Indeed, NTA analysis measures the hydrodynamic diameter which relates to the Brownian motion of the particle in a fluid that can be influenced by temperature, ionic strength of the media and the surface structure of the particle. Moreover, SEM measurement requires a dehydration step which can also influence the size of the particle.

Functional analyses of hNSC-EVs were focused on their immunomodulatory phenotype. We first implemented an in vitro model employing murine microglial cells (BV2) treated with LPS to mimic neuroinflammation [25, 53]. Indeed, microglial cells, the monophagocitic cells of the CNS, respond to an inflammatory stimulus inducing the expression of iNOS which leads to an increase in NO levels; the dysregulation of this pathway is involved in neurodegenerative and neuroinflammatory diseases [54]. The uptake of hNSC-EVs induced a reduction in NO levels associated with a decrease in iNOS protein expression, supporting the notion that hNSC-EVs can reduce the inflammatory burden in CNS. Data in the literature demonstrated that EVs isolated from hiPS-Derived Neural Stem Cell Cultures (hiPSC-NSC-EVs) reduce inflammation in human microglia by transporting miR-21 and pentraxin 3 [18, 20]. The identification of miR21 among the cargo of hNSC-EVs raises the possibility that a similar mechanism might be involved in the observed functional effects. However, further experiments are needed to draw a definitive conclusion. It is important to note that EVs may exert cross-species activities. For example, intranasally administered hNSC-EVs were demonstrated to be potent inhibitors of microglia activation in a mouse model of early-stage Alzheimer’s disease [18–20]. In addition, EVs derived from hAFSC-EVs can be taken up by murine cDC2, resulting in their reprogramming toward a tolerogenic phenotype and a subsequent decrease in the production of pro-inflammatory mediators [16]. These reprogrammed cDC2s not only secrete anti-inflammatory cytokines but also release secondary EVs capable of reaching the CNS. Together, this dual mechanism promotes the attenuation of experimental autoimmune encephalomyelitis (EAE), supports the restoration of immune homeostasis, and mitigates neuronal degeneration and autoimmune responses [16].

Given the close link between neuroinflammation and peripheral inflammation [2, 41, 55], we functionally characterized hNSC-EVs using the human monocytic cell line THP-1. We focused specifically on inflammasome activation, due to its central role in neuroinflammatory processes [56]. The NLRP3 inflammasome is a key mediator of neuroinflammation, sensing cellular stress and promoting the release of IL-1β and IL-18, along with the induction of pyroptotic cell death [56]. This inflammatory cascade contributes to BBB disruption, neuronal dysfunction, and chronic neuroinflammation [57]. Our results showed that hNSC-EVs inhibit NLRP3 inflammasome activation in THP-1 monocytic cells. A likely mechanism underlying this anti-inflammatory effect is the autonomous production of adenosine. Indeed, hNSC-EVs can autonomously synthesize ATP and express CD73 (ecto 5’ nucleotidase) which is responsible for its dephosphorylation. Moreover, the pre-treatment with the adenosine A2a receptor antagonist prevented hNSC-EVs effects on inflammasome activation. These findings support the notion that EVs exert immunomodulatory effects not only through their cargo but also through their intrinsic metabolic activity [58, 59].

Our findings highlight the therapeutic potential of hNSC-EVs in modulating neuroinflammation via a dual, cell-type-specific mechanism. In microglial cells, hNSC-EVs reduce NO production, likely through cargo-mediated signaling; in monocytic cells, they suppress NLRP3 inflammasome activation through adenosine-A2a receptor signaling. These results are supported by studies recognizing adenosine receptors as important orchestrators of inflammation and key regulators of tissue homeostasis [12, 60].

The dual mode of action of hNSC-EVs underscores the context-dependent versatility of EVs, revealing their capacity to engage distinct molecular pathways across different immune cell types to elicit a coordinated anti-inflammatory response.

Our study has some limitations. We performed an in-depth characterization of the proteome of hNSCs, but future studies will need to investigate also the molecular cargo of the hNSC-EVs with the aim of understanding which proteins, RNAs or lipids are secreted within the vesicles and may be responsible for the biological effects towards target cells. Also, the physiological effect of hNSCs interplay with target cells would be of interest; in this scenario assessing the functional outcome in co-culture settings should be objective of future studies. Additionally, all our experiments were performed using in vitro models, that do not allow the study of EVs interactions within physiological systems. Therefore, a characterization of hNSC-EVs effect in animal models of human neurological diseases is needed, to fully comprehend the best dosing, administration route, and possible effects in the periphery or in the CNS [16]. It will also be mandatory to explore more robust isolation methods than ultracentrifugation, to improve the yield, purity and standardize EVs isolation for future clinical applications.

## Conclusions

In summary, our findings indicate that GMP-manufactured hNSCs retain a strong staminal signature and preserve the ability to differentiate. We also showed that hNSCs secrete EVs with anti-inflammatory and immunomodulatory properties. Notably, the effects of hNSC-EVs can take place through two distinct mechanisms: one that depends on cellular uptake and cargo transfer of and another that operates in an uptake-independent fashion suggesting that EVs released by hNSCs may affect inflammation either through direct interactions with target cells or by altering the surrounding microenvironment. Altogether, our results support the notion that stem cell therapies can provide beneficial effects not only caused by the cell themselves, which can differentiate in the transplantation site or migrate to repair the damage, but also by stem cell secreted EVs, which can affect residing cells by transferring their cargo or by modulating the microenvironment composition. A groundbreaking scenario envisions EVs not just as an alternative to stem cell therapy but rather as a means to enhance their efficacy; pretreatment with EVs prior to stem cell transplant could significantly boost treatment outcomes, marking a pioneering advancement in regenerative medicine.

## Supplementary Information

Below is the link to the electronic supplementary material.

Supplementary Material 1.

Supplementary Material 2.

Supplementary Material 3.

Supplementary Material 4.

Supplementary Material 5.

Supplementary Material 6.

Supplementary Material 7.

Supplementary Material 8.

## Data Availability

The data sets for the research presented here are available from the corresponding author on reasonable request. All additional files are included in the manuscript.

## Abbreviations

Alix: Programmed cell death 6-interacting protein
ALPL: Alkaline phosphatase
ALS: Amyotrophyc Lateral Sclerosis
BBB: Blood brain barrier
CD39: Ectonucleoside triphosphate diphosphohydrolase 1
cDC2: Conventional dendritic cells
CNS: Central nervous system
COX IV: Cytochrome c oxidase subunit IV
CXCR4 (CD184): C-X-C chemokine receptor type 4
CSF: Cerebrospinal fluid
DCX: Doublecortin
DIA: Data independent acquisition
EVs: Extracellular vesicles
GAP43: Growth associated protein 43
GAPDH: Glyceraldehyde 3-phosphate dehydrogenase
GFAP: Glial Fibrillary Acidic Protein
GMP: Good manufacturing practice
hAFSC-EVs: EVs derived from human amniotic fluid stem cell lines
hiPSC-NSC-EVs: Human induced pluripotent stem cell-derived neural stem cell extracellular vesicles
hNSC-EVs: Human neural stem cell-derived extracellular vesicles
hNSCs: Human neural stem cells
IHC: Immunohistochemistry
Olig2: Oligodendrocyte transcription factor 2
iNOS: Inducible nitric oxide synthase
KLF4: Krüppel-like factor 4
iPSC: Induced Pluripotent Stem Cell
LPS: Lipopolysaccharide
MAP2: Microtubule-associated protein 2
MS: Multiple sclerosis
MSI1: RNA-binding protein Musashi homolog 1
NCAN: Neurocan core protein
NES: Nestin
NLRP3: NLR family pyrin domain containing 3
NO: Nitric oxide
NSCs: Neural stem cells
NSE: Neuron-specific enolase
NT5E (CD73): 5’-Nucleotidase ecto
NTA: Nanoparticle tracking analysis
OCT4: Octamer-binding transcription factor 4
OLIG1: Oligodendrocyte transcription factor 1
PLP1: Proteolipid protein 1
PODXL: Podocalyxin
PROM1: Prominin-1
S100B: S100 calcium-binding protein B
SEM: Scanning electron microscopy
SLC1A2: Solute carrier family 1 member 2
SOX2: SRY-box transcription factor 2
TSG101: Tumor susceptibility gene 101

## References

[1] Taupin P. Adult neural stem cells, neurogenic niches, and cellular therapy. Stem Cell Rev. 2006;2:213–9. 17625257 10.1007/s12015-006-0049-0

[2] Kokaia Z, Martino G, Schwartz M, Lindvall O. Cross-talk between neural stem cells and immune cells: the key to better brain repair? Nat Neurosci. 2012;15:1078–87. 22837038 10.1038/nn.3163

[3] Mazzini L, Gelati M, Profico DC, Sgaravizzi G, Projetti Pensi M, Muzi G, et al. Human neural stem cell transplantation in ALS: initial results from a phase I trial. J Transl Med. 2015;13:17. 25889343 10.1186/s12967-014-0371-2 PMC4359401

[4] Mazzini L, Gelati M, Profico DC, Sorarù G, Ferrari D, Copetti M, et al. Results from phase I clinical trial with intraspinal injection of neural stem cells in amyotrophic lateral sclerosis: A Long-Term outcome. Stem Cells Transl Med. 2019;8:887–97. 31104357 10.1002/sctm.18-0154 PMC6708070

[5] Leone MA, Gelati M, Profico DC, Gobbi C, Pravatà E, Copetti M, et al. Phase I clinical trial of intracerebroventricular transplantation of allogeneic neural stem cells in people with progressive multiple sclerosis. Cell Stem Cell. 2023;30:1597–e16098. 38016468 10.1016/j.stem.2023.11.001

[6] Genchi A, Brambilla E, Sangalli F, Radaelli M, Bacigaluppi M, Furlan R, et al. Neural stem cell transplantation in patients with progressive multiple sclerosis: an open-label, phase 1 study. Nat Med. 2023;29:75–85. 36624312 10.1038/s41591-022-02097-3 PMC9873560

[7] Glass JD, Boulis NM, Johe K, Rutkove SB, Federici T, Polak M, et al. Lumbar intraspinal injection of neural stem cells in patients with amyotrophic lateral sclerosis: results of a phase I trial in 12 patients. Stem Cells. 2012;30:1144–51. 22415942 10.1002/stem.1079

[8] Zhang K, Cheng K. Stem cell-derived exosome versus stem cell therapy. Nat Reviews Bioeng. 2023;1–2. 10.1038/s44222-023-00064-2 PMC10092910 37359776

[9] Welsh JA, Goberdhan DCI, O’Driscoll L, Buzas EI, Blenkiron C, Bussolati B, et al. Minimal information for studies of extracellular vesicles (MISEV2023): from basic to advanced approaches. J Extracell Vesicles. 2024;13:e12404. 38326288 10.1002/jev2.12404 PMC10850029

[10] Garcia NA, Moncayo-Arlandi J, Sepulveda P, Diez-Juan A. Cardiomyocyte exosomes regulate glycolytic flux in endothelium by direct transfer of GLUT transporters and glycolytic enzymes. Cardiovasc Res. 2016;109:397–408. 26609058 10.1093/cvr/cvv260

[11] Romani R, Talesa VN, Antognelli C. The glyoxalase system is a novel cargo of amniotic fluid Stem-Cell-Derived extracellular vesicles. Antioxid (Basel). 2022;11. 10.3390/antiox11081524 PMC9405222 36009243

[12] Mezzasoma L, Bellezza I, Orvietani P, Manni G, Gargaro M, Sagini K et al. Amniotic fluid stem cell-derived extracellular vesicles are independent metabolic units capable of modulating inflammasome activation in THP-1 cells. FASEB J [Internet]. 2022 [cited 2025 Jul 11];36. 10.1096/fj.202101657R 35218567

[13] Gurunathan S, Kang M-H, Song H, Kim NH, Kim J-H. The role of extracellular vesicles in animal reproduction and diseases. J Anim Sci Biotechnol. 2022;13:62. 35681164 10.1186/s40104-022-00715-1 PMC9185900

[14] Han J, Zhang X, Kang L, Guan J. Extracellular vesicles as therapeutic modulators of neuroinflammation in alzheimer’s disease: a focus on signaling mechanisms. J Neuroinflammation. 2025;22:120. 40281600 10.1186/s12974-025-03443-1 PMC12023694

[15] Mezzasoma L, Talesa VN, Costanzi E, Bellezza I. Natriuretic peptides regulate prostate cells inflammatory behavior: potential novel anticancer agents for prostate cancer. Biomolecules. 2021;11(6):794. 34070682 10.3390/biom11060794 PMC8228623

[16] Manni G, Gargaro M, Ricciuti D, Fontana S, Padiglioni E, Cipolloni M, et al. Amniotic fluid stem cell-derived extracellular vesicles educate type 2 conventional dendritic cells to rescue autoimmune disorders in a multiple sclerosis mouse model. J Extracell Vesicles. 2024;13:e12446. 38844736 10.1002/jev2.12446 PMC11156524

[17] Chen J, Tian C, Xiong X, Yang Y, Zhang J. Extracellular vesicles: new horizons in neurodegeneration. EBioMedicine. 2025;113:105605. 40037089 10.1016/j.ebiom.2025.105605 PMC11925178

[18] Upadhya R, Madhu LN, Rao S, Shetty AK. Proficiency of extracellular vesicles from hiPSC-Derived neural stem cells in modulating Proinflammatory human microglia: role of Pentraxin-3 and miRNA-21-5p. Front Mol Neurosci. 2022;15:845542. 35656007 10.3389/fnmol.2022.845542 PMC9152457

[19] Madhu LN, Kodali M, Upadhya R, Rao S, Somayaji Y, Attaluri S, et al. Extracellular vesicles from human-induced pluripotent stem cell-derived neural stem cells alleviate Proinflammatory cascades within disease-associated microglia in alzheimer’s disease. J Extracell Vesicles. 2024;13:e12519. 39499013 10.1002/jev2.12519 PMC11536387

[20] Garcia G, Pinto S, Ferreira S, Lopes D, Serrador MJ, Fernandes A et al. Emerging role of miR-21-5p in Neuron-Glia dysregulation and exosome transfer using multiple models of alzheimer’s disease. Cells. 2022;11. 10.3390/cells11213377 PMC9655962 36359774

[21] Gelati M, Profico DC, Ferrari D, Vescovi AL. Culturing and expansion of clinical grade neural stem cells from the fetal human central nervous system. Methods Mol Biol. 2022;2389:57–66. 34558001 10.1007/978-1-0716-1783-0_5

[22] Núñez FJ, Mendez FM, Garcia-Fabiani MB, Pardo J, Edwards M, Lowenstein PR et al. Evaluation of biomarkers in glioma by immunohistochemistry on Paraffin-Embedded 3D glioma neurosphere cultures. J Vis Exp. 2019. 10.3791/58931 PMC6352905 30688315

[23] Théry C, Witwer KW, Aikawa E, Alcaraz MJ, Anderson JD, Andriantsitohaina R, et al. Minimal information for studies of extracellular vesicles 2018 (MISEV2018): a position statement of the international society for extracellular vesicles and update of the MISEV2014 guidelines. J Extracell Vesicles. 2018;7:1535750. 30637094 10.1080/20013078.2018.1535750 PMC6322352

[24] Rider MA, Hurwitz SN, Meckes DG. ExtraPEG: A polyethylene Glycol-Based method for enrichment of extracellular vesicles. Sci Rep. 2016;6:23978. 27068479 10.1038/srep23978 PMC4828635

[25] Grottelli S, Amoroso R, Macchioni L, D’Onofrio F, Fettucciari K, Bellezza I et al. Acetamidine-Based iNOS inhibitors as molecular tools to counteract inflammation in BV2 microglial cells. Molecules. 2020;25. 10.3390/molecules25112646 PMC7321217 32517272

[26] Davidescu M, Mezzasoma L, Fettucciari K, Pascucci L, Pariano M, Di Michele A, et al. Cardiolipin-mediated Temporal response to hydroquinone toxicity in human retinal pigmented epithelial cell line. Biochim Biophys Acta Mol Cell Res. 2023;1870:119554. 37524263 10.1016/j.bbamcr.2023.119554

[27] Romani R, Pirisinu I, Calvitti M, Pallotta MT, Gargaro M, Bistoni G, et al. Stem cells from human amniotic fluid exert immunoregulatory function via secreted indoleamine 2,3-dioxygenase1. J Cell Mol Med. 2015;19:1593–605. 25783564 10.1111/jcmm.12534 PMC4511357

[28] Macchioni L, Chiasserini D, Mezzasoma L, Davidescu M, Orvietani PL, Fettucciari K et al. Crosstalk between Long-Term sublethal oxidative stress and detrimental inflammation as potential drivers for Age-Related retinal degeneration. Antioxid (Basel). 2020;10. 10.3390/antiox10010025 PMC7823845 33383836

[29] Kessner D, Chambers M, Burke R, Agus D, Mallick P. ProteoWizard: open source software for rapid proteomics tools development. Bioinformatics. 2008;24:2534–6. 18606607 10.1093/bioinformatics/btn323 PMC2732273

[30] Demichev V, Messner CB, Vernardis SI, Lilley KS, Ralser M. DIA-NN: neural networks and interference correction enable deep proteome coverage in high throughput. Nat Methods. 2020;17:41–4. 31768060 10.1038/s41592-019-0638-x PMC6949130

[31] Yu G, Wang L-G, Han Y, He Q-Y. ClusterProfiler: an R package for comparing biological themes among gene clusters. OMICS. 2012;16:284–7. 22455463 10.1089/omi.2011.0118 PMC3339379

[32] Liberzon A, Birger C, Thorvaldsdóttir H, Ghandi M, Mesirov JP, Tamayo P. The molecular signatures database (MSigDB) hallmark gene set collection. Cell Syst. 2015;1:417–25. 26771021 10.1016/j.cels.2015.12.004 PMC4707969

[33] Profico DC, Gelati M, Ferrari D, Sgaravizzi G, Ricciolini C, Projetti Pensi M et al. Human neural stem Cell-Based drug product: clinical and nonclinical characterization. Int J Mol Sci. 2022;23. 10.3390/ijms232113425 PMC9653902 36362211

[34] Komitova M, Mattsson B, Johansson BB, Eriksson PS. Enriched environment increases neural stem/progenitor cell proliferation and neurogenesis in the subventricular zone of stroke-lesioned adult rats. Stroke. 2005;36:1278–82. 15879324 10.1161/01.STR.0000166197.94147.59

[35] Qi H, Pei D. The magic of four: induction of pluripotent stem cells from somatic cells by Oct4, Sox2, Myc and Klf4. Cell Res. 2007;17:578–80. 17632550 10.1038/cr.2007.59

[36] Horánszky A, Shashikadze B, Elkhateib R, Lombardo SD, Lamberto F, Zana M, et al. Proteomics and disease network associations evaluation of environmentally relevant bisphenol A concentrations in a human 3D neural stem cell model. Front Cell Dev Biol. 2023;11:1236243. 37664457 10.3389/fcell.2023.1236243 PMC10472293

[37] Červenka J, Tylečková J, Kupcová Skalníková H, Vodičková Kepková K, Poliakh I, Valeková I, et al. Proteomic characterization of human neural stem cells and their secretome during in vitro differentiation. Front Cell Neurosci. 2020;14:612560. 33584205 10.3389/fncel.2020.612560 PMC7876319

[38] Yuan SH, Martin J, Elia J, Flippin J, Paramban RI, Hefferan MP, et al. Cell-surface marker signatures for the isolation of neural stem cells, glia and neurons derived from human pluripotent stem cells. PLoS ONE. 2011;6:e17540. 21407814 10.1371/journal.pone.0017540 PMC3047583

[39] Zhang J, Jiao J. Molecular biomarkers for embryonic and adult neural stem cell and neurogenesis. Biomed Res Int. 2015;2015:727542. 26421301 10.1155/2015/727542 PMC4569757

[40] Barnett RE, Conklin DJ, Ryan L, Keskey RC, Ramjee V, Sepulveda EA, et al. Anti-inflammatory effects of miR-21 in the macrophage response to peritonitis. J Leukoc Biol. 2016;99:361–71. 26382295 10.1189/jlb.4A1014-489R PMC6608009

[41] Singh J, Habean ML, Panicker N. Inflammasome assembly in neurodegenerative diseases. Trends Neurosci. 2023;46:814–31. 37633753 10.1016/j.tins.2023.07.009 PMC10530301

[42] Mezzasoma L, Schmidt-Weber CB, Fallarino F. In vitro study of TLR4-NLRP3-Inflammasome activation in innate immune response. Methods Mol Biol. 2023;2700:163–76. 37603180 10.1007/978-1-0716-3366-3_9

[43] Wilson DM, Cookson MR, Van Den Bosch L, Zetterberg H, Holtzman DM, Dewachter I. Hallmarks of neurodegenerative diseases. Cell. 2023;186:693–714. 36803602 10.1016/j.cell.2022.12.032

[44] Lupia M, Angiolini F, Bertalot G, Freddi S, Sachsenmeier KF, Chisci E, et al. CD73 regulates stemness and Epithelial-Mesenchymal transition in ovarian Cancer-Initiating cells. Stem Cell Rep. 2018;10:1412–25. 10.1016/j.stemcr.2018.02.009 PMC5998305 29551673

[45] Tan K, Zhu H, Zhang J, Ouyang W, Tang J, Zhang Y, et al. CD73 expression on mesenchymal stem cells dictates the reparative properties via its Anti-Inflammatory activity. Stem Cells Int. 2019;2019:8717694. 31249602 10.1155/2019/8717694 PMC6525959

[46] Palmqvist L, Glover CH, Hsu L, Lu M, Bossen B, Piret JM, et al. Correlation of murine embryonic stem cell gene expression profiles with functional measures of pluripotency. Stem Cells. 2005;23:663–80. 15849174 10.1634/stemcells.2004-0157

[47] Pluchino S, Zanotti L, Rossi B, Brambilla E, Ottoboni L, Salani G, et al. Neurosphere-derived multipotent precursors promote neuroprotection by an Immunomodulatory mechanism. Nature. 2005;436:266–71. 16015332 10.1038/nature03889

[48] Pluchino S, Quattrini A, Brambilla E, Gritti A, Salani G, Dina G, et al. Injection of adult neurospheres induces recovery in a chronic model of multiple sclerosis. Nature. 2003;422:688–94. 12700753 10.1038/nature01552

[49] Musiał-Wysocka A, Kot M, Majka M. The pros and cons of mesenchymal stem Cell-Based therapies. Cell Transpl. 2019;28:801–12. 10.1177/0963689719837897 PMC6719501 31018669

[50] Fernandez-Muñoz B, Garcia-Delgado AB, Arribas-Arribas B, Sanchez-Pernaute R. Human neural stem cells for Cell-Based medicinal products. Cells. 2021;10. 10.3390/cells10092377 PMC8469920 34572024

[51] Willis CM, Nicaise AM, Peruzzotti-Jametti L, Pluchino S. The neural stem cell secretome and its role in brain repair. Brain Res. 2020;1729:146615. 31863730 10.1016/j.brainres.2019.146615

[52] Xavier CPR, Caires HR, Barbosa MAG, Bergantim R, Guimarães JE, Vasconcelos MH. The role of extracellular vesicles in the hallmarks of cancer and drug resistance. Cells. 2020;9. 10.3390/cells9051141 PMC7290603 32384712

[53] Zhang W, Xiao D, Mao Q, Xia H. Role of neuroinflammation in neurodegeneration development. Signal Transduct Target Ther. 2023;8:267. 37433768 10.1038/s41392-023-01486-5 PMC10336149

[54] Calabrese V, Mancuso C, Calvani M, Rizzarelli E, Butterfield DA, Stella AMG. Nitric oxide in the central nervous system: neuroprotection versus neurotoxicity. Nat Rev Neurosci. 2007;8:766–75. 17882254 10.1038/nrn2214

[55] Süβ P, Lana AJ, Schlachetzki JCM. Chronic peripheral inflammation: a possible contributor to neurodegenerative diseases. Neural Regen Res. 2021;16:1711–4. 33510059 10.4103/1673-5374.306060 PMC8328777

[56] Heneka MT, Kummer MP, Stutz A, Delekate A, Schwartz S, Vieira-Saecker A, et al. NLRP3 is activated in Alzheimer’s disease and contributes to pathology in APP/PS1 mice. Nature. 2013;493:674–8. 23254930 10.1038/nature11729 PMC3812809

[57] Voet S, Srinivasan S, Lamkanfi M, van Loo G. Inflammasomes in neuroinflammatory and neurodegenerative diseases. EMBO Mol Med. 2019;11. 10.15252/emmm.201810248 PMC6554670 31015277

[58] Iraci N, Gaude E, Leonardi T, Costa ASH, Cossetti C, Peruzzotti-Jametti L, et al. Extracellular vesicles are independent metabolic units with asparaginase activity. Nat Chem Biol. 2017;13:951–5. 28671681 10.1038/nchembio.2422 PMC5563455

[59] Ronquist KG, Sanchez C, Dubois L, Chioureas D, Fonseca P, Larsson A, et al. Energy-requiring uptake of prostasomes and PC3 cell-derived exosomes into non-malignant and malignant cells. J Extracell Vesicles. 2016;5:29877. 26955882 10.3402/jev.v5.29877 PMC4783432

[60] Rabbani P, Ramkhelawon B, Cronstein BN. Adenosine metabolism and receptors in aging of the skin, musculoskeletal, immune and cardiovascular systems. Ageing Res Rev. 2025;106:102695. 39971100 10.1016/j.arr.2025.102695 PMC11960428

