# Supplementary material for: Molecular and functional characterization of GMP-manufactured neural stem cells and their extracellular vesicles for innovative therapeutic applications
Source: Stem Cell Res Ther. 2026 Jan 9;17:74. doi: 10.1186/s13287-026-04904-x (PMC12882627; doi:10.1186/s13287-026-04904-x)
Supplement: Supplementary file 1 — Supplementary Material 1. [file 13287_2026_4904_MOESM1_ESM.docx]

**Table 1.** Antibody list

| Acronym | Antibody | Diluition | Company | Catalog number |
| --- | --- | --- | --- | --- |
| Primary antibodies | | | | |
| Alix | ALG-2-interacting protein X (mAb) | 1:200^†^ | Santa Cruz Biotechnology | sc-53540 |
| Calnexin | Calnexin (mAb) | 1:1000^†^ | Cell Signaling Technology | #2679 |
| CD73 | 5′-nucleotidase (pAb) | 1:1000^†^ | Elabscience | E-AB-60564 |
| CD81 | Tetraspanin-28 (mAb) | 1:500^†^ | Santa Cruz Biotechnology | sc-166029 |
| COX4 | Cytochrome c oxidase (mAb) | 1:500^†^ | Santa Cruz Biotechnology | sc-376731 |
| Csp-1 | Caspase-1 (pAb) | 1:1000^‡^ | Cell Signaling Technology | #2225 |
| GalC | Galactocerebrosidase | 1:200 ^¶^ | Sigma-Aldrich | MAB342 |
| GAPDH | Glyceraldehyde-3-Phosphate Dehydrogenase (mAb) | 1:500^†^ | Santa Cruz Biotechnology | sc-32233 |
| GFAP | Glial Fibrillary Acidic Protein (EP672Y) (mAb) | Prediluted ready-to-use | Cell Marque | 258R |
| GFAP | Glial Fibrillary Acidic Protein | 1:500 ^¶^ | Sigma-Aldrich | MAB3402 |
| iNOS | Inducible nitric oxyde synthase (pAb) | 1:250^†^ | Santa Cruz Biotechnology | sc-650 |
| Ki-67 | Anti-Ki-67 (30-9) (mAb) | Prediluted ready-to-use | Roche | 790-4286 |
| KLF4 | anti‑Human KLF4 Antibody | 1:10^§^ | LSBio | LS‑C96469 |
| NSE | Neuron-specific enolase (MRQ-55) (mAb) | Prediluted ready-to-use | Cell Marque | 306M |
| OCT4 | Octamer binding transcription factor 4 (mAb) | 10 µl ^§^ | Biolegend | 653710 |
| Olig2 | oligodendrocyte lineage transcription factor 2 (211F1.1) (mAb) | Prediluted ready-to-use | Cell Marque | 387M |
| SOX2 | SRY-Box Transcription Factor 2 (pAb) | 1:500^†^ | ABclonal | A11501 |
| Tsg101 | Tumor susceptibility gene 101 (mAb) | 1:200^†^ | Santa Cruz Biotechnology | sc-7964 |
| TUBB-III | beta-tubulin III | 1:10000 ^¶^ | BioLegend | 802001 |
| β-actin | β-actin (pAb) | 1:1000^‡^ | Santa Cruz Biotechnology | sc-1616 |
| Secondary antibodies | | | | |
|  | Anti-Mouse IgG-HRP linked | 1:4000^†, ‡^ | Cell Signaling Technology | #7076 |
|  | Anti-Rabbit IgG-HRP linked | 1:5000^†, ‡^ | Cell Signaling Technology | #7074 |
|  | Goat anti-Rabbit IgG (H+L) Cross-Adsorbed Secondary Antibody, Alexa Fluor™ 488 | 1:1000^¥^ | Thermo Fisher Scientific | A-11008 |
|  | Goat anti-Mouse IgG (H+L) Cross-Adsorbed Secondary Antibody, Alexa Fluor™ 546 | 1:1000^¥^ | Thermo Fisher Scientific | A-11003 |
|  | PE Goat anti-mouse Ig | 1:400^§^ | BioLegend | 405307 |

^†^ Dilution buffer: 5% skim milk in TBS-T

^‡^ Dilution buffer: Rotiblock

^§^ Antibody for FACS analysis

^¶^ Dilution buffer: 10% NGS (Normal Goat Serum) in PBS

^¥^ Dilution buffer: PBS
