# Supplementary material for: Molecular and functional characterization of GMP-manufactured neural stem cells and their extracellular vesicles for innovative therapeutic applications
Source: Stem Cell Res Ther. 2026 Jan 9;17:74. doi: 10.1186/s13287-026-04904-x (PMC12882627; doi:10.1186/s13287-026-04904-x)
Supplement: Supplementary file 2 — Supplementary Material 2. [file 13287_2026_4904_MOESM2_ESM.docx]

**Table 2.** PCR primer sequences

| Name | Forward sequences | Reverse sequences |
| --- | --- | --- |
| ALP | 5’ACCGCCACCGCCTACCTG3’ | 5’GTCACAATGCCCACAGATTTCCC3 |
| SOX2 | 5’AAGTAGTTTGCTGCCTCTTTAAG3’ | 5’GCTTCCCTCCTCCTCTGG3’ |
| OCT-4a | 5’ACGACCATCTGCCGCTTTG3’ | 5’GTTGCCTCTCACTCGGTTCTC3’ |
| KLF4 | 5’ACGGCTGTGGATGGAAATTC3’ | 5’ATGTGTAAGGCGAGGTGGTC3’ |
| GAPDH | 5' TGTAAAACGACGGCCAGT3’ | 5' CAGGAAACAGCTATGACC3’ |
| miR21-5p stem loop | 5′ctcaactggtgtcgtggagtcggcaattcagttgagtcaacatc3′ |  |
| miR21-5p | 5′ACACTCCAGCTGGGTAGCTTATCAGACTGA3 | 5′tggtgtcgtggagtcg3′ |
