# Supplementary figures and images for: Molecular and functional characterization of GMP-manufactured neural stem cells and their extracellular vesicles for innovative therapeutic applications
Source: Stem Cell Res Ther. 2026 Jan 9;17:74. doi: 10.1186/s13287-026-04904-x (PMC12882627; doi:10.1186/s13287-026-04904-x)

Supplementary to figure 5

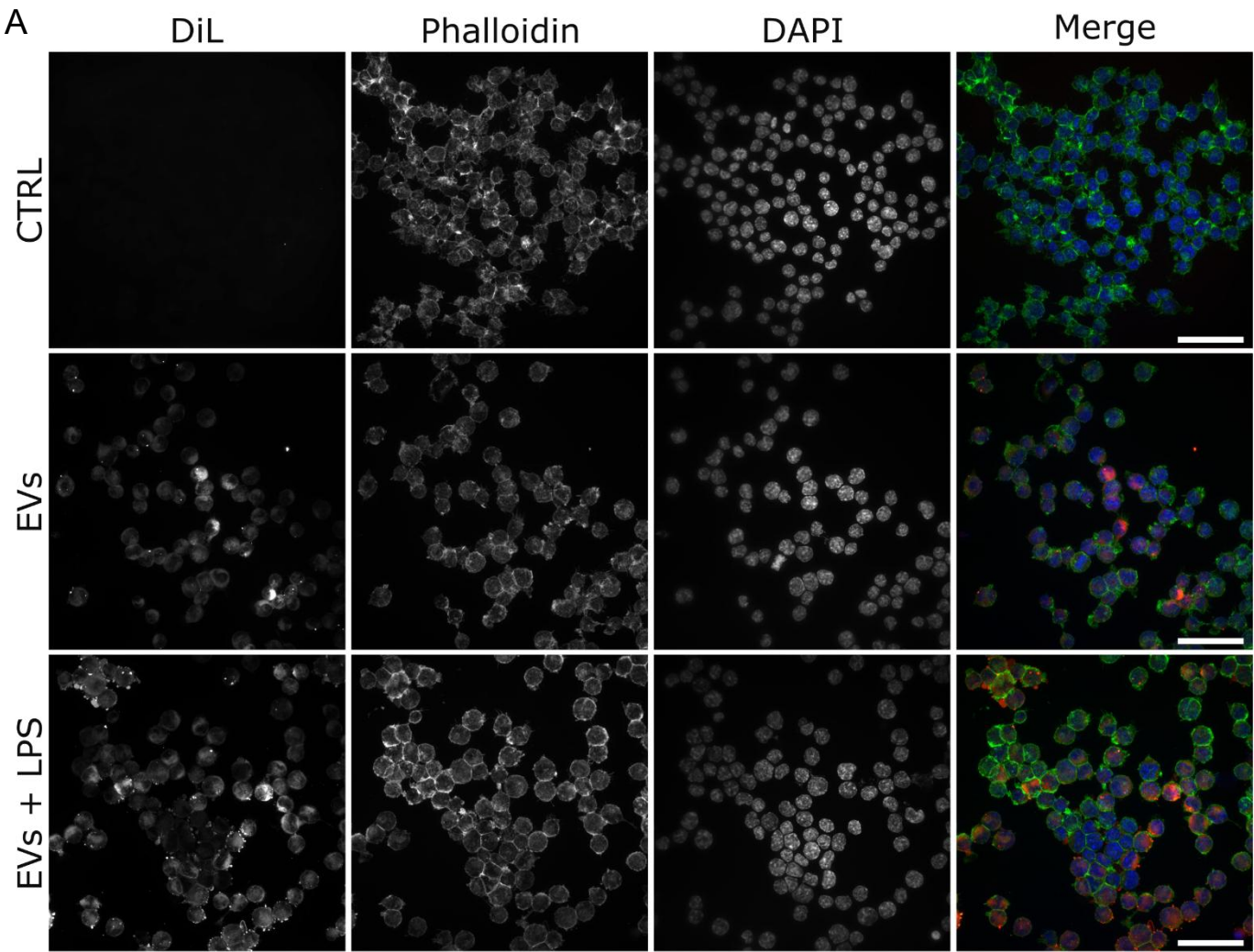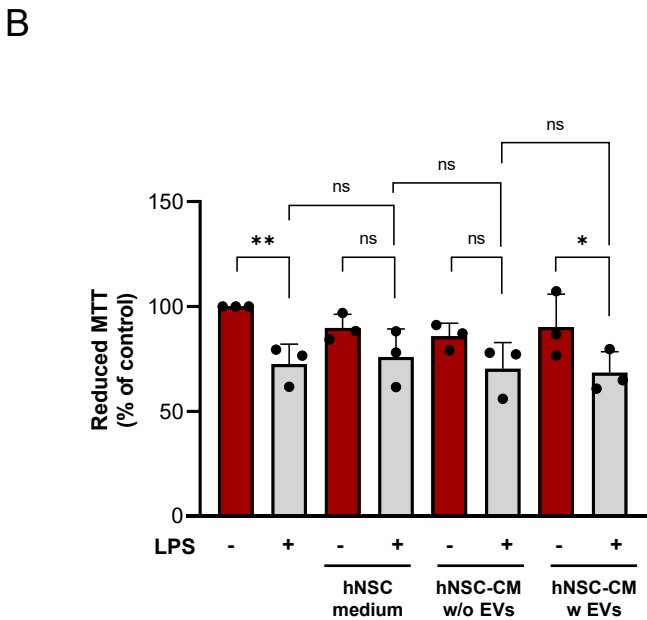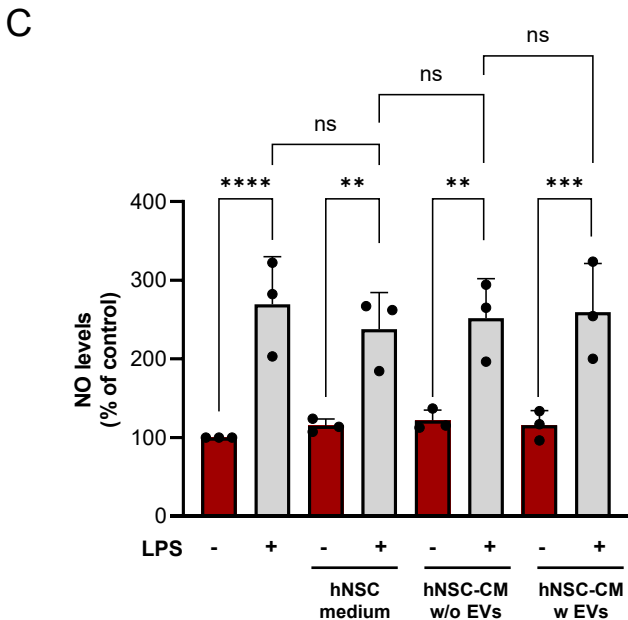

|                 | DiI | Phalloidin | DAPI | Merge |
|-----------------|-----|------------|------|-------|
| CTRL            |     |            |      |       |
| EVs             |     |            |      |       |
| EVs + LPS + ATP |     |            |      |       |

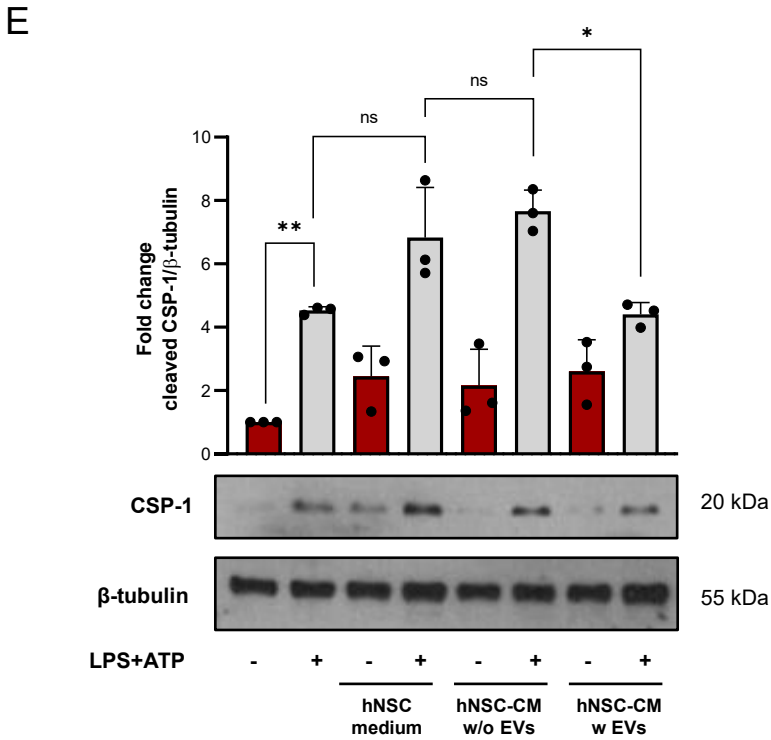

Supplement: Supplementary file 6 — Supplementary Material 6. [file 13287_2026_4904_MOESM6_ESM.pdf]
