# Supplementary material for: Molecular and functional characterization of GMP-manufactured neural stem cells and their extracellular vesicles for innovative therapeutic applications
Source: Stem Cell Res Ther. 2026 Jan 9;17:74. doi: 10.1186/s13287-026-04904-x (PMC12882627; doi:10.1186/s13287-026-04904-x)
Supplement: Supplementary file 7 — Supplementary Material 7. [file 13287_2026_4904_MOESM7_ESM.pdf]

Molecular and functional characterization of GMP-manufactured neural stem cells and their extracellular vesicles for innovative therapeutic applications

Martina Guzzetti[1], Letizia Mezzasoma[1], Davide Chiasserini[1,2], Lara Macchioni[1], Magdalena Davidescu[1], Alessandro di Michele[2, 3], Marco Gargaro[4], Nicola Di-Iacovo[1], Giorgia Manni[1, 2], Gianmarco Muzi[6], Ilaria Proietti[6], Giuseppina Bevacqua[5], Eleonora Becattini[5], Carlo Conti[5], Vincenzo Nicola Talesa[1], Rita Romani[1, 2], Ilaria Bellezza[1, 2], Valentina Grespi[5,6].

[1]Department of Medicine and Surgery, University of Perugia, Perugia, Italy.
[2]Extracellular Vesicles network (EV-net) of the University of Perugia, Perugia, Italy.
[3]Department of Physics and Geology, University of Perugia, Perugia, Italy.
[4]Department of Pharmaceutical Sciences, University of Perugia, Perugia, Italy.
[5]Department of Neuroscience, Neurosurgery Unit, Santa Maria Hospital, Terni, Italy.
[6]Laboratorio Cellule Staminali, Cell Factory e Biobanca, Santa Maria Hospital, Terni, Italy.

Martina Guzzetti and Letizia Mezzasoma contributed equally to the work
Valentina Grespi and Ilaria Bellezza share senior authorship, and both are corresponding Authors

ADDITIONAL FILE

Additional_file_1.docx Table 1. Antibody list

Additional_file_2.docx Table 2. PCR primer sequences

Additional_file_3.xlsx Table 3. List of stem cell markers used for overlap analysis.

Additional_file_4.xlsx Table 4. List of genes specific for the main brain cell types.

Additional_file_5.xlsx Table 5. Identified proteins in three replicates of neural stem cells.

Additional_file_6.xlsx Supplementary to figure 5 and 6

ADDITIONAL FILE LEGEND

Additional file 6. Supplementary to figure 5 and 6
A, D) hNSC-EVs uptake by BV2 cells and THP1 cells. Cells were treated with DiL-labeled hNSC-EVs for 1h and then exposed for 24h with or without LPS (BV2) or for 1h with or without LPS+ATP (THP1), phalloidin was used for actin filaments staining and nuclei were counterstained with Hoechst 33258. The merged images show DiL staining in red, phalloidin in green and nuclei in blue; a 50 µm scale bar is reported. BV2 (B-C) and THP1 (E) were pre-treated for 1h with non-conditioned medium (hNSC-medium), EV-depleted conditioned medium (hNSC-CM w/o EVs) and conditioned media with EVs (hNSC-CM w/EVs) and then exposed to LPS for 24h (B-C) and LPS and ATP for 1h (E) as described in material and methods. B) Cell viability, detected by MTT assay (absorbance of control cells = 2.4 ± 0.3 was assumed as 100%). Data represent mean ± SD of *n* = 3 independent experiments performed in quadruplicate; C) NO production, detected by Griess reagent (absorbance of control cells = 0.053 ± 0.002 was assumed as 100%). Data represent mean ± SD of *n* = 3 independent experiments performed in quadruplicate; E) THP1 cell lysates were immunoblotted for Caspase-1. The blots were re-probed with mouse anti-β-tubulin, to confirm equal loading. Representative western blots images are shown. Histogram represents densitometric quantification and indicates the mean ± SD of *n* = 3 independent experiments.
