# Supplementary material for: Molecular and functional characterization of GMP-manufactured neural stem cells and their extracellular vesicles for innovative therapeutic applications
Source: Stem Cell Res Ther. 2026 Jan 9;17:74. doi: 10.1186/s13287-026-04904-x (PMC12882627; doi:10.1186/s13287-026-04904-x)

Supplementary whole blots for revision only.
Images used in the manuscript figures are highlighted in yellow.

EXP 1_EVs

hSNC-EVs | hSNC-EVs | hSNC

Calnexin
90kDa

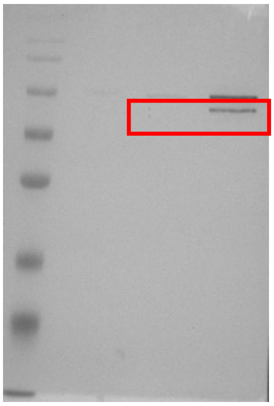

hSNC-EVs | hSNC-EVs | hSNC

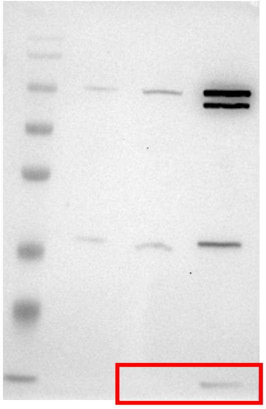

Cox IV
17kDa

hSNC-EVs | hSNC-EVs | hSNC

ALIX
95kDa

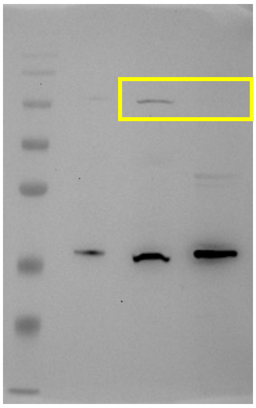

hSNC-EVs | hSNC-EVs | hSNC

GAPDH
37kDa

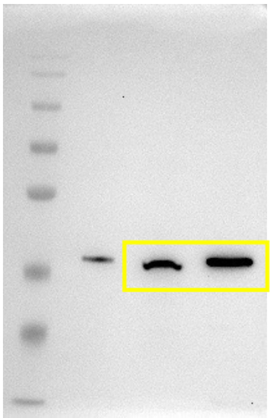

hSNC-EVs | hSNC | THP1

CD73
70kDa

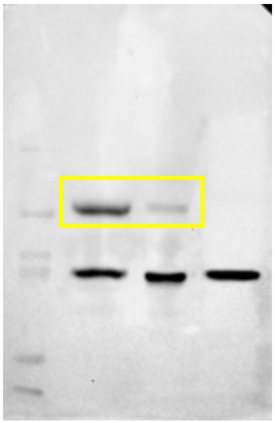

hSNC | hSNC-EVs | hSNC | hSNC-EVs

TSG101
45kDa

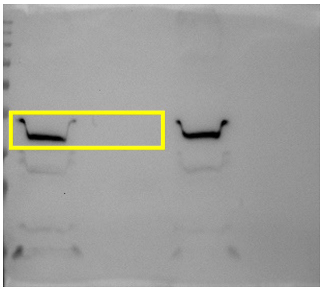

hSNC | hSNC-EVs | hSNC | hSNC-EVs

CD81
26kDa

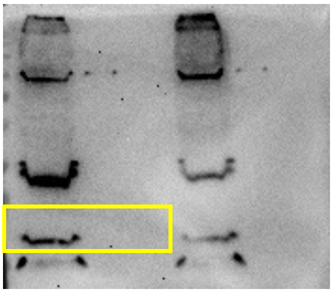

Some blots were used for multiple Abs

EXP 2_EVs

hSNC-EVs hSNC

Cox IV 17kDa

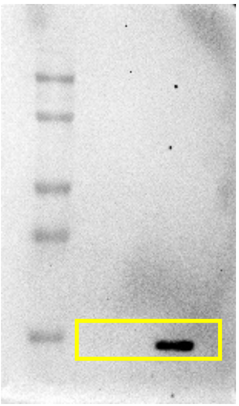

hSNC-EVs hSNC

ALIX 95kDa

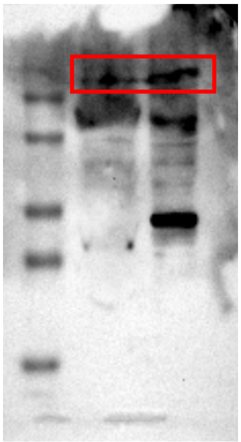

hSNC-EVs hSNC

GAPDH 37kDa

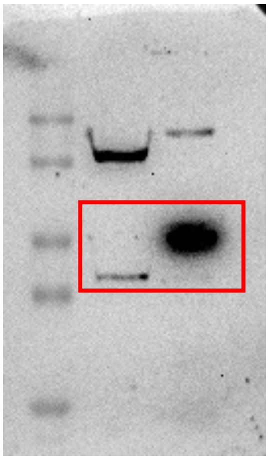

hSNC-EVs hSNC

Calnexin 90kDa

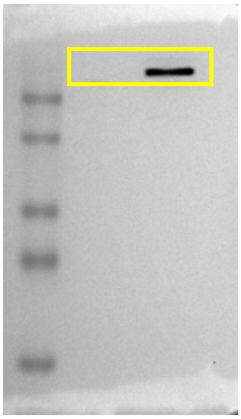

hSNC hSNC-EVs

CD81 26kDa

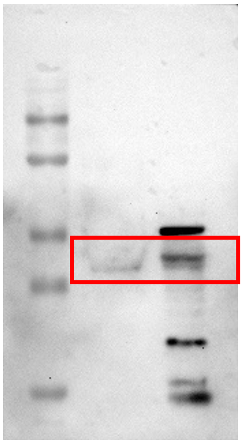

hSNC-EVs hSNC

CD73 70kDa

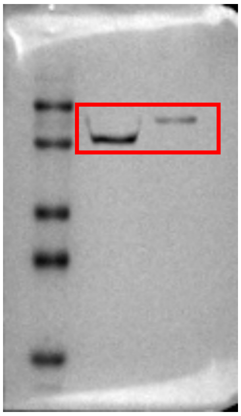

Some blots were used for multiple Abs

Exp 1 – 2_hNSC

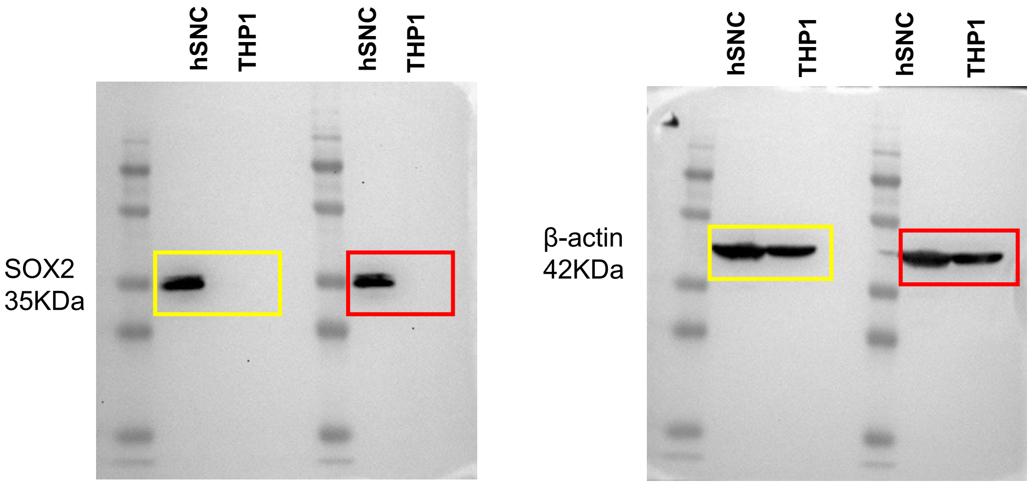

Exp 3_hNSC

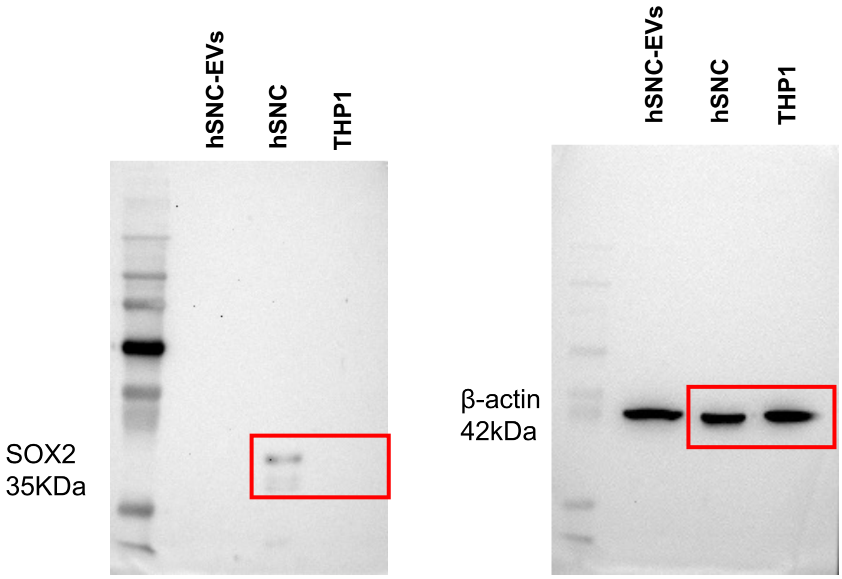

EXP 1-2-3-4_BV2

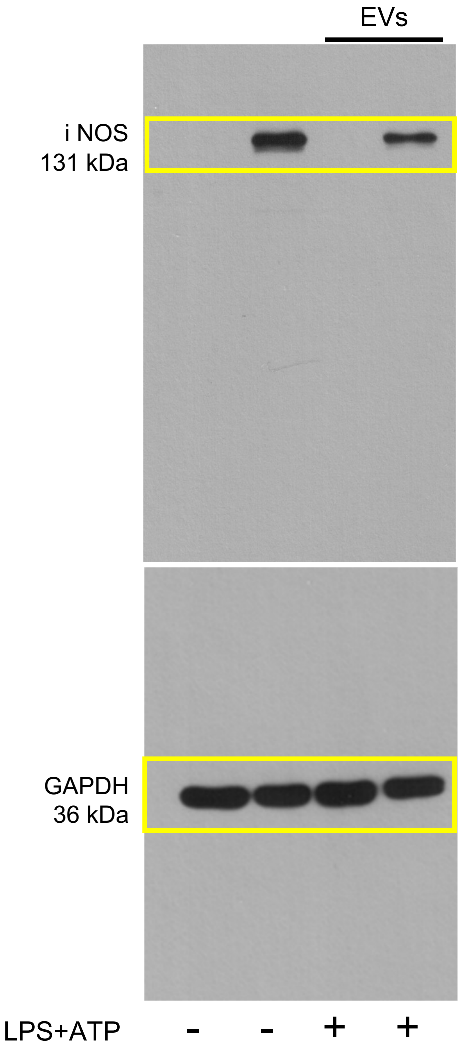
EVs
i NOS
131 kDa
GAPDH
36 kDa
LPS+ATP - - + +

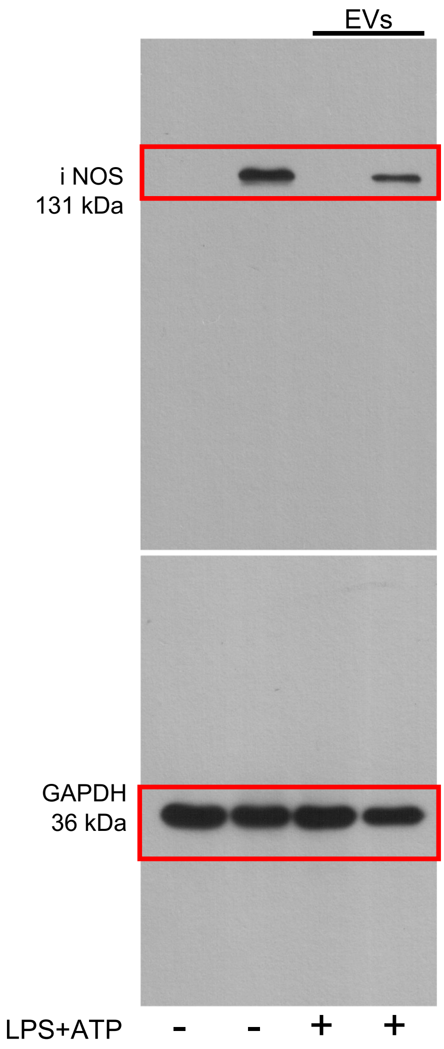
EVs
i NOS
131 kDa
GAPDH
36 kDa
LPS+ATP - - + +

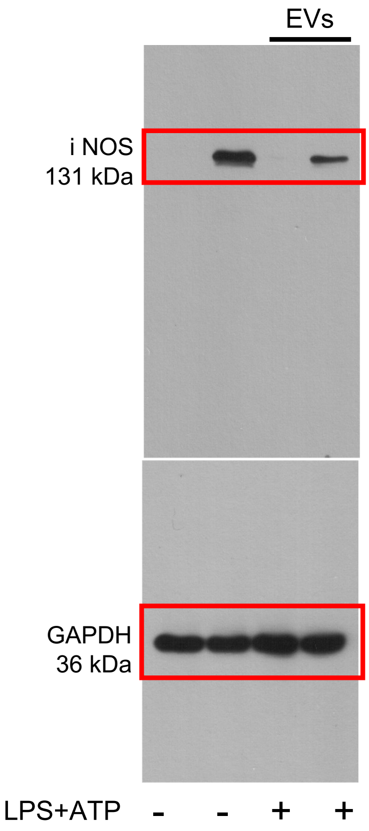
EVs
i NOS
131 kDa
GAPDH
36 kDa
LPS+ATP - - + +

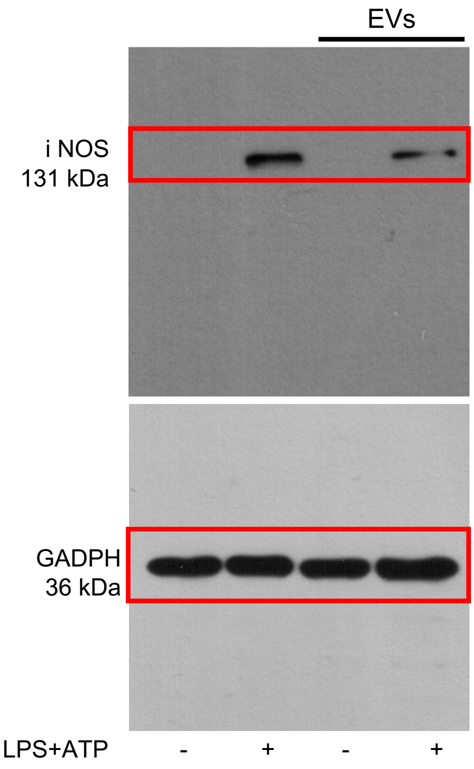
EVs
i NOS
131 kDa
GADPH
36 kDa
LPS+ATP - + - +

EXP 1-2-3-4_THP1

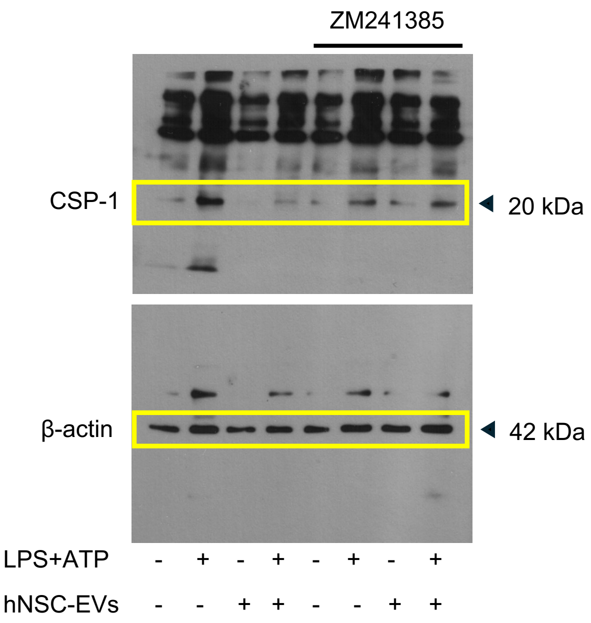

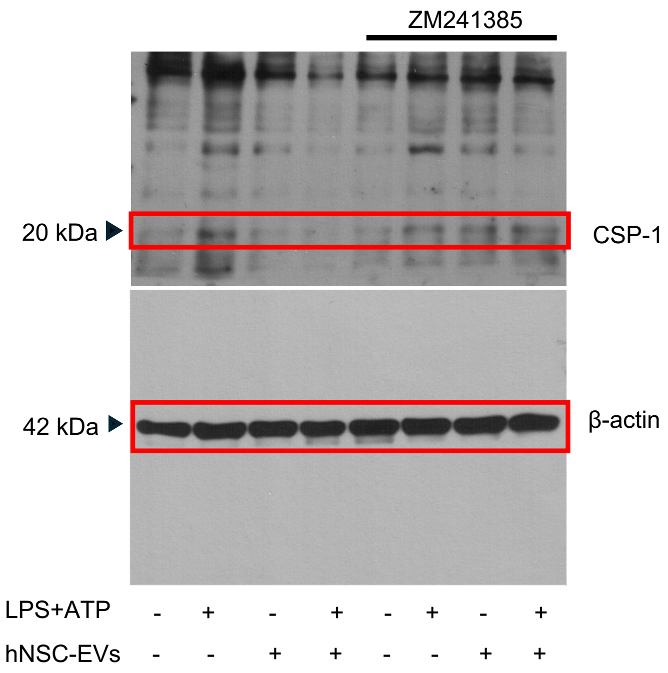

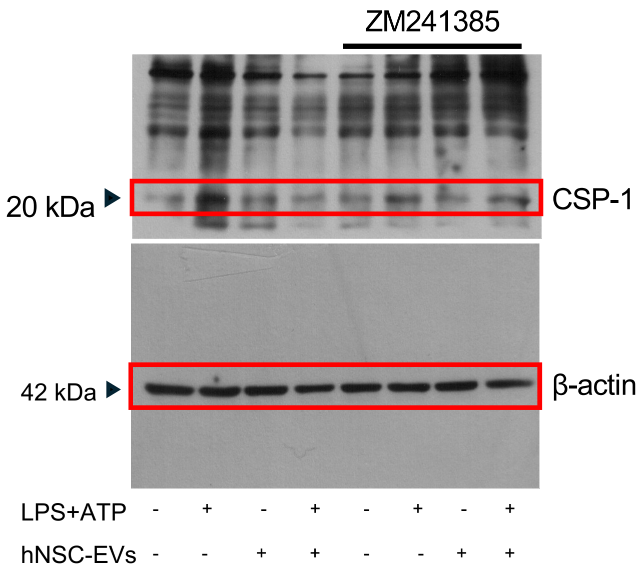

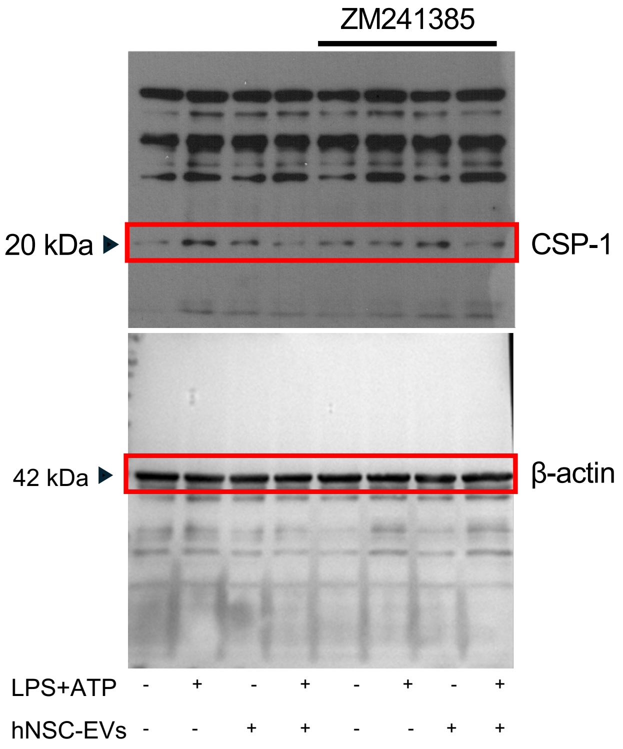

EXP 1-2-3_THP1_medium

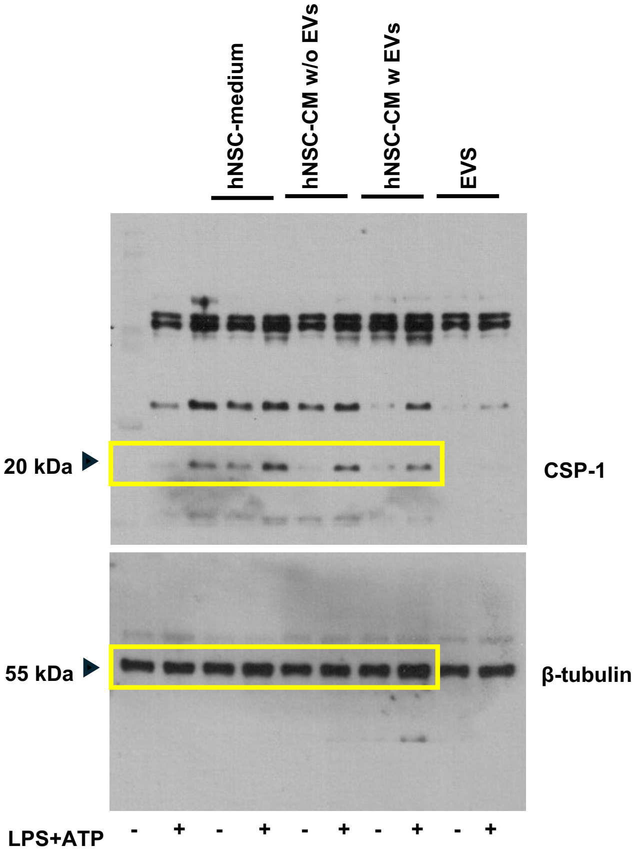

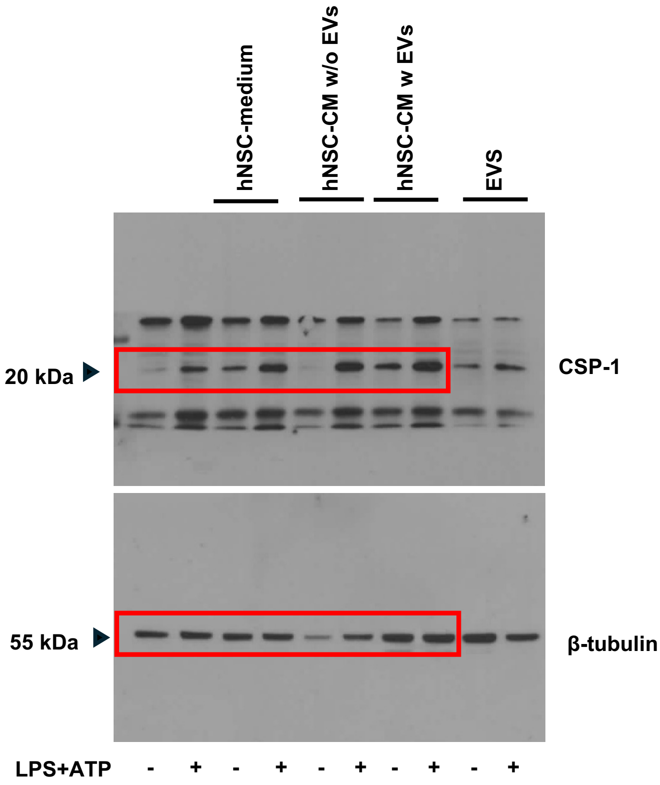

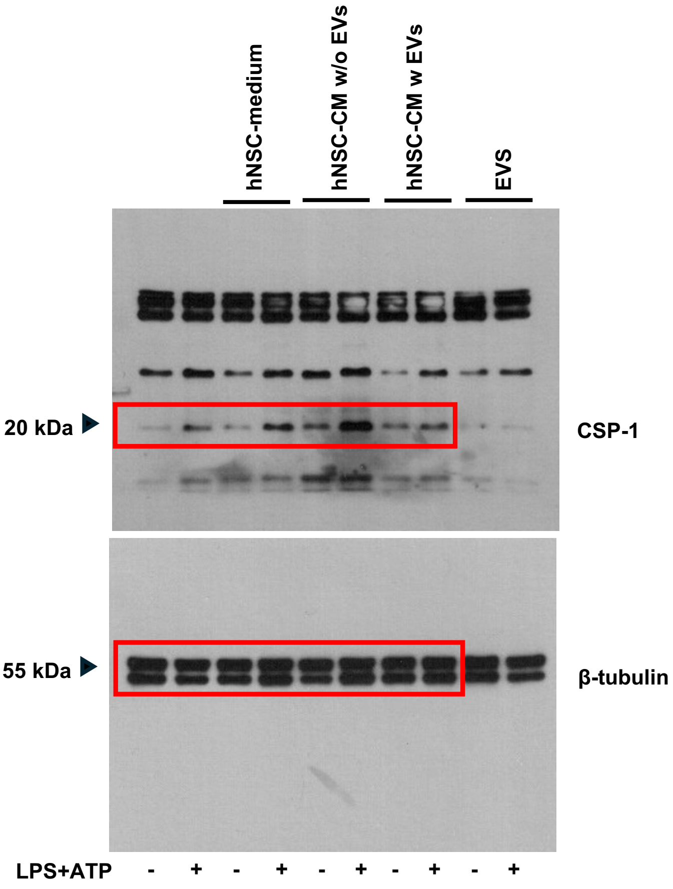

Supplement: Supplementary file 8 — Supplementary Material 8. [file 13287_2026_4904_MOESM8_ESM.pdf]
